# Evolutionary history of Tibetans inferred from whole-genome sequencing

**DOI:** 10.1371/journal.pgen.1006675

**Published:** 2017-04-27

**Authors:** Hao Hu, Nayia Petousi, Gustavo Glusman, Yao Yu, Ryan Bohlender, Tsewang Tashi, Jonathan M. Downie, Jared C. Roach, Amy M. Cole, Felipe R. Lorenzo, Alan R. Rogers, Mary E. Brunkow, Gianpiero Cavalleri, Leroy Hood, Sama M. Alpatty, Josef T. Prchal, Lynn B. Jorde, Peter A. Robbins, Tatum S. Simonson, Chad D. Huff

**Affiliations:** 1 Department of Epidemiology, University of Texas MD Anderson Cancer Center, Houston, Texas, United States of America; 2 Nuffield Department of Medicine, University of Oxford, Oxford, United Kingdom; 3 Institute for Systems Biology, Seattle, Washington, United States of America; 4 Department of Anthropology, University of Utah, Salt Lake City, Utah, United States of America; 5 Department of Medicine, University of Utah School of Medicine and George E. Wahlin Veterans Administration Medical Center, Salt Lake City, Utah, United States of America; 6 Department of Human Genetics, University of Utah, Salt Lake City, Utah, United States of America; 7 Department of Molecular and Cellular Therapeutics, The Royal College of Surgeons in Ireland, Dublin, Ireland; 8 Skaggs School of Pharmacy and Pharmaceutical Science, UC San Diego, La Jolla, California, United States of America; 9 Department of Physiology, Anatomy and Genetics, University of Oxford, Oxford, United Kingdom; 10 Department of Medicine, Division of Physiology, University of California San Diego, La Jolla, California, United States of America; University of Pennsylvania, UNITED STATES

## Abstract

The indigenous people of the Tibetan Plateau have been the subject of much recent interest because of their unique genetic adaptations to high altitude. Recent studies have demonstrated that the Tibetan *EPAS1* haplotype is involved in high altitude-adaptation and originated in an archaic Denisovan-related population. We sequenced the whole-genomes of 27 Tibetans and conducted analyses to infer a detailed history of demography and natural selection of this population. We detected evidence of population structure between the ancestral Han and Tibetan subpopulations as early as 44 to 58 thousand years ago, but with high rates of gene flow until approximately 9 thousand years ago. The CMS test ranked *EPAS1* and *EGLN1* as the top two positive selection candidates, and in addition identified *PTGIS*, *VDR*, and *KCTD12* as new candidate genes. The advantageous Tibetan *EPAS1* haplotype shared many variants with the Denisovan genome, with an ancient gene tree divergence between the Tibetan and Denisovan haplotypes of about 1 million years ago. With the exception of *EPAS1*, we observed no evidence of positive selection on Denisovan-like haplotypes.

## Author summary

The Tibetan population has been residing on high plateau for thousands of years and developed unique adaptation to the local environment. To investigate the demographic history of Tibetans and search for possible adaptive genetic variants, we performed whole-genome sequencing of 27 Tibetan individuals. We found evidence of genetic separation between Han and Tibetans around since 44 and 58 thousand years ago; however, these two populations maintained a high rate of gene flow until 9 thousand years ago. In addition to replicating two previously discovered candidate genes (*EGLN1 and EPAS1*) for high altitude adaptation, we also found three new candidate genes, including *PTGIS*, *VDR* and *KCTD12*. We confirmed the high similarity of *EPAS1* gene region between Tibetans and Denisovans, but did not detect any evidence of high altitude adaptation from Denisovan gene alleles otherwise.

## Introduction

Adaptation to high altitude is among the most notable examples of natural selection in our species. Tibetans exhibit an exceptional capacity to compensate for decreased oxygen availability (hypoxia), UV exposure, cold, and limited food sources as exemplified by continuous habitation at greater than 4000 meters above sea level on the Qinghai-Tibetan Plateau over several millennia[1–4].

Many candidate genes have been highlighted as likely contributors to Tibetans’ trans-generational success at high altitude[5–9]. These findings are largely based upon the overlap of *a priori* genes, specifically those involved in hypoxia tolerance, and genomic footprints of adaptation identified in patterns within single nucleotide polymorphism (SNP) microarrays[10–12] or exome sequencing data[13]. In a few cases, putatively adaptive tagging variants in hypoxia inducible factor (HIF) pathway genes (*EPAS1*[10,13], *EGLN1* and *PPARA*[12]) are associated with relatively lower hemoglobin concentration exhibited by many Tibetans at altitude. With the exception of the *EGLN1* locus[14,15], however, precise functional variants within these genomic regions are unknown.

Recent advances in whole-genome sequencing (WGS) technology provide progress toward identifying the functional variants that underlie high-altitude adaptation. Rather than focusing on a relatively small portion of the genome using candidate gene approaches, WGS selection analyses often have sufficient power to comprehensively interrogate the genome in an unbiased manner. Moreover, WGS offers the opportunity to perform exhaustive searches for adaptive variation, yielding complete surveys of non-protein coding regulatory, conserved, and structural variants possibly tagged or missed in microarray analyses. WGS data are also important from an evolutionary standpoint, providing unbiased insights into long-standing questions regarding adaptive processes, including the role of adaptive introgression, as recently revealed through the targeted sequence comparisons between Tibetans and the Denisovan genome at the *EPAS1* gene locus[16].

To identify regions contributing to high-altitude adaptation and regions with archaic introgressions with high resolution, we performed a comprehensive genomic analysis of WGS data in 27 Tibetans[17]. We estimated the demographic history of Tibetans using MSMC and *∂a∂i*, and performed the Composite of Multiple Signals test for fine-mapping loci and variants with recent selective sweep. We also estimated genome-wide levels of archaic admixture, and generated a fine-scale map of Denisovan-like introgression in Tibetans.

## Results

### Global genomic analysis and demographic history estimate

We conducted principal components analysis (PCA) on all SNVs with observed non-reference allele frequency greater than 5% among the combined samples of Tibetans and five 1000 Genomes Project[18] populations (Peruvian, Punjabi, Chinese, Yoruba and European) (Supplementary Methods). Tibetans and Han Chinese were closely clustered in the first 4 principal components (S1A and S1B Fig) similar to our previous observation[12], but were clearly differentiated along 5^th^ principal component (S1C Fig). Similarly, ADMIXTURE analysis[19] (Supplementary Methods) on the same six populations shows that when the number of ancestral populations (K) was set to less than 6, Tibetans and Han Chinese were genetically similar. However, when K was set to 6 or greater, these two populations exhibited distinct genetic profiles (S2 Fig). The estimated proportion of Tibetan genetic ancestry was greater than 99% for 19 out of the 27 Tibetan individuals. For the remaining Tibetan individuals, the average estimated proportion of Tibetan genetic ancestry was 83.1%. For the demographic history analysis, we excluded Tibetan individuals with less than 99% Tibetan genetic ancestry. F_ST_ between Tibetans and Chinese based on our SNV data was 0.0148 using the Hudson estimator[20] and 0.0149 using the Weir and Cockerham estimator [21], similar to our previous analyses based on SNP microarray data[22], which is slightly higher than the F_ST_ between Chinese and Japanese populations but lower than the F_ST_ between pairs of HapMap populations from different continents [23].

We next annotated the functional impact of SNVs that are frequent in Tibetans but uncommon in other populations. We selected all germline SNVs with an alternative allele frequency above 10% in Tibetans but below 1% among Yoruba, Han Chinese, and Europeans from the 1000 Genomes Project. In total, we identified 10,702 such SNVs, among which there were 65 nonsynonymous variants that occurred within 58 genes (S1 Table). We predicted the impact of these protein-altering SNVs on genome functions using PolyPhen-2 and the Conservation-controlled Amino Acid Substitution Matrix (CASM) in VAAST 2.0[24]. Of the 65 nonsynonymous variants, Polyphen-2 predicted 12 to be functional and CASM predicted a distinct set of 3 variants to be functional. One of the variants, Chr2:233244223 A->C (p.T104P; all genomic coordinates in this manuscript are relative to GRCh37 reference sequence), is in the gene *ALPP* with an observed frequency of 0.14% in dbSNP and 14.81% in Tibetans. *ALPP* encodes an alkaline phosphatase, which is mainly expressed in placental and endometrial tissues. Rare nonsynonymous variants in *ALPP* have previously been associated with decreased risk of spontaneous abortion and in vitro fertilization failure[25].

**Fig 1:**
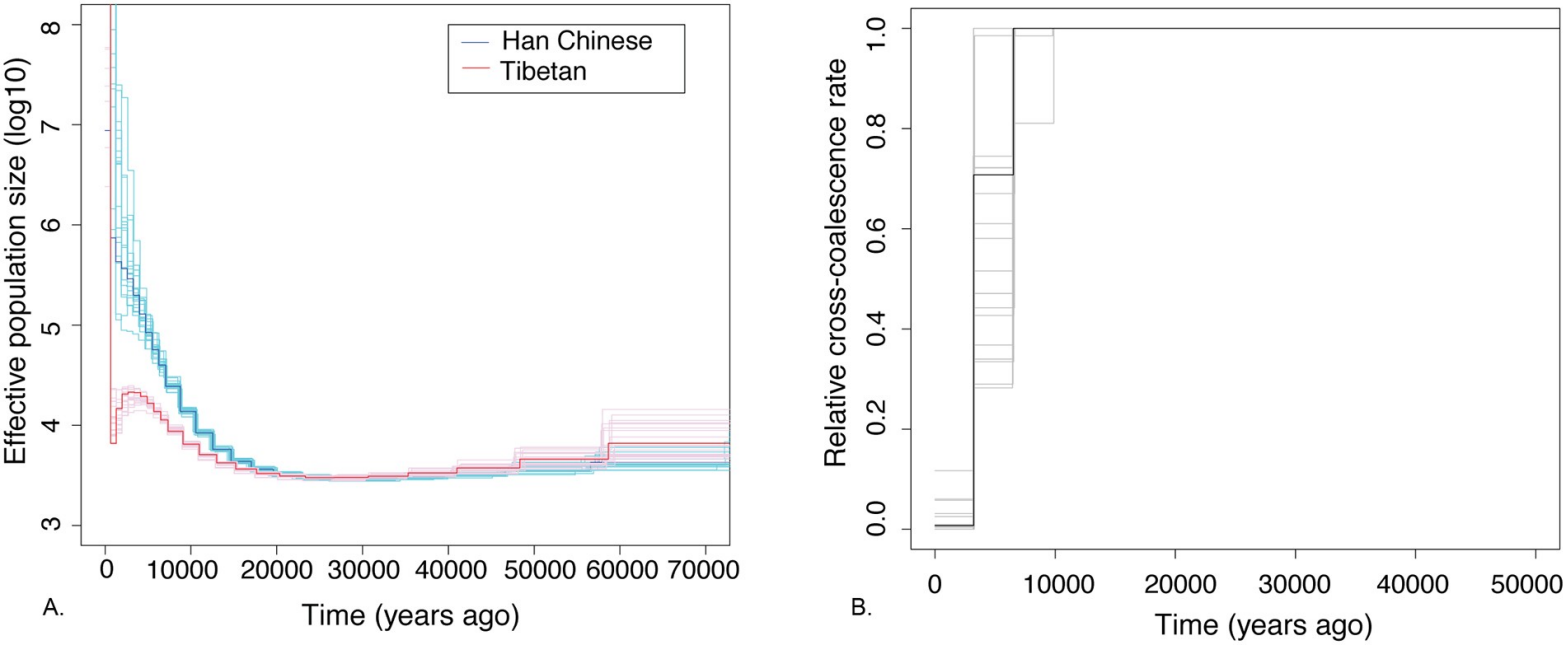
MSMC estimate of population histories for Tibetans and Han Chinese. (A) The effective population size as the function of time in the past for Tibetans (red) and Han (blue), estimated using 4 Han and 4 Tibetan genomes with greater than 99% corresponding genetic ancestries. (B) The relative coalescence rate between Tibetans and Han as the function of time in the past, estimated using 1 Han and 1 Tibetan genomes with greater than 99% corresponding genetic ancestries. Solid color indicates the estimates from the actual data, and the corresponding lighter colors indicate the estimates from 20 bootstrapped datasets.

**Fig 2:**
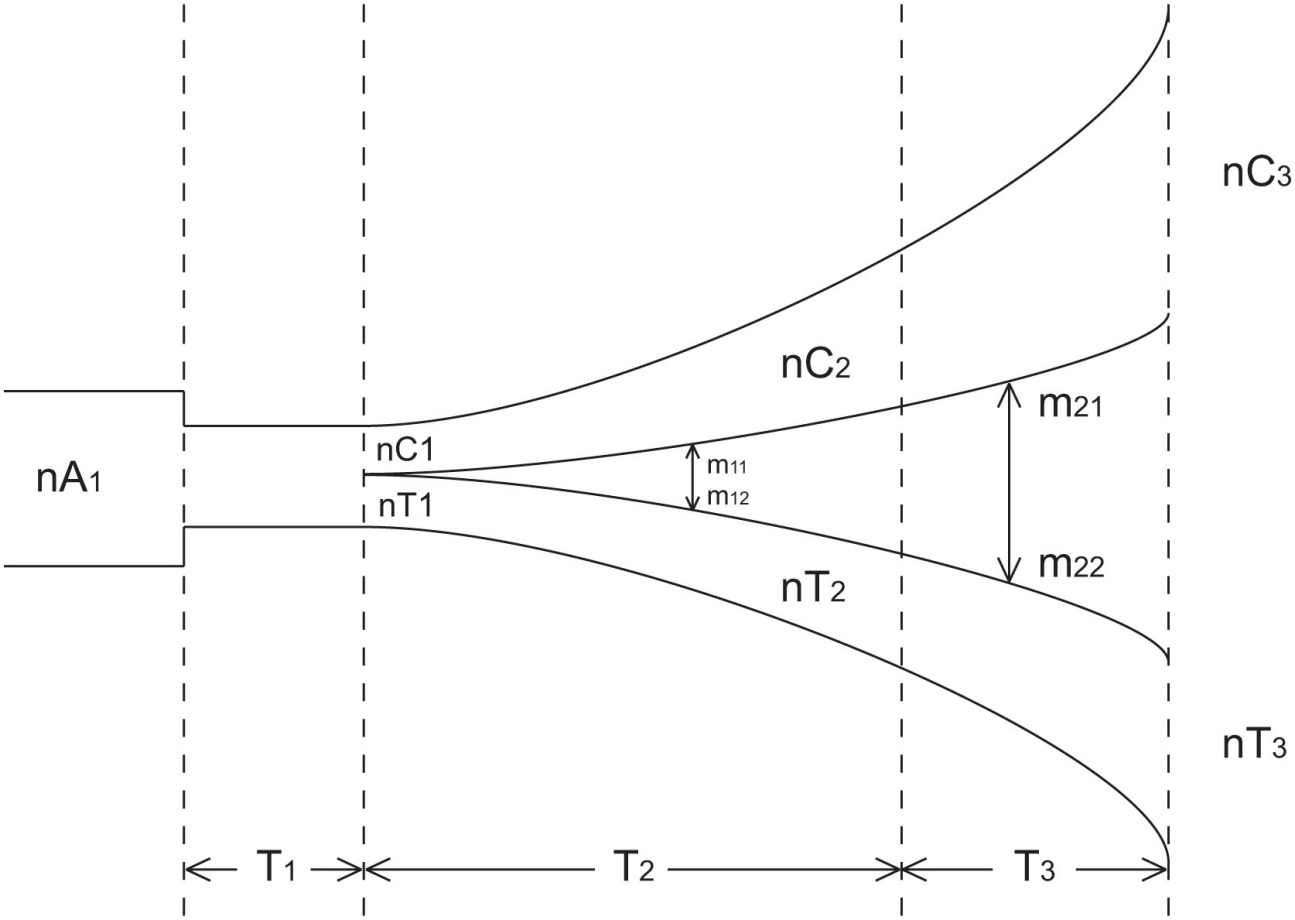
Illustration of the best-fitting ∂a∂i model.

We inferred the demographic history of Tibetans using the multiple sequential Markovian Coalescent (MSMC)[26] (Fig 1) and *∂a∂i*[27] (Fig 2; Table 1) (Supplementary Methods). MSMC estimated that the relative cross coalescence rate (a measurement of the amount of gene flow between two populations) between Han and Tibetans fell below 80% around 7 kya (bootstrap 95% CI: 3kya to 10kya). We then derived a more detailed demographic model using *∂a∂i*, which predicted that the initial divergence between Han and Tibetan was much earlier, at 54kya (bootstrap 95% C.I 44 kya to 58 kya). However, for the first 45ky, the two populations maintained substantial gene flow (6.8x10^-4^ and 9.0x10^-4^ per generation per chromosome). After 9.4 kya (bootstrap 95% C.I 8.6 kya to 11.2 kya), the gene flow rate dramatically dropped (1.3x10^-11^ and 4x10^-7^ per generation per chromosome), which is consistent with the estimate from MSMC. F_ST_ predicted by the best-fitting demographic model in *∂a∂i* produced estimates consistent with observed F_ST_ (0.0147 using Hudson’s F_ST_ estimator and 0.0148 using the Weir and Cockerham’s estimator).

**Table 1:** Point estimate and 95% confidence interval of the parameters of the best fitting ∂a∂i model (see Fig 2 for the explanation of the parameters).

	point estimate	lower 95% CI	upper 95% CI
nA1	12804	12619	13445
nC1	500	412	607
nC2	75203	70047	86703
nC3	1326988	490163	5940902
nT1	2445	2098	5251
nT2	13292	10292	14033
nT3	77743	63131	148702
T1 (years)	6355	4365	38991
T2 (years)	44588	35784	47330
T3 (years)	9419	8573	11166
m11 (/gen/chrom)	6.8E-04	5.8E-04	9.0E-04
m12 (/gen/chrom)	9.0E-04	8.0E-04	1.0E-03
m21 (/gen/chrom)	1.3E-11	8.6E-12	1.9E-11
m22 (/gen/chrom)	4.0E-07	2.3E-18	6.5E-06

### Whole-genome positive selection scans in the Tibetan genome

**Fig 3:**
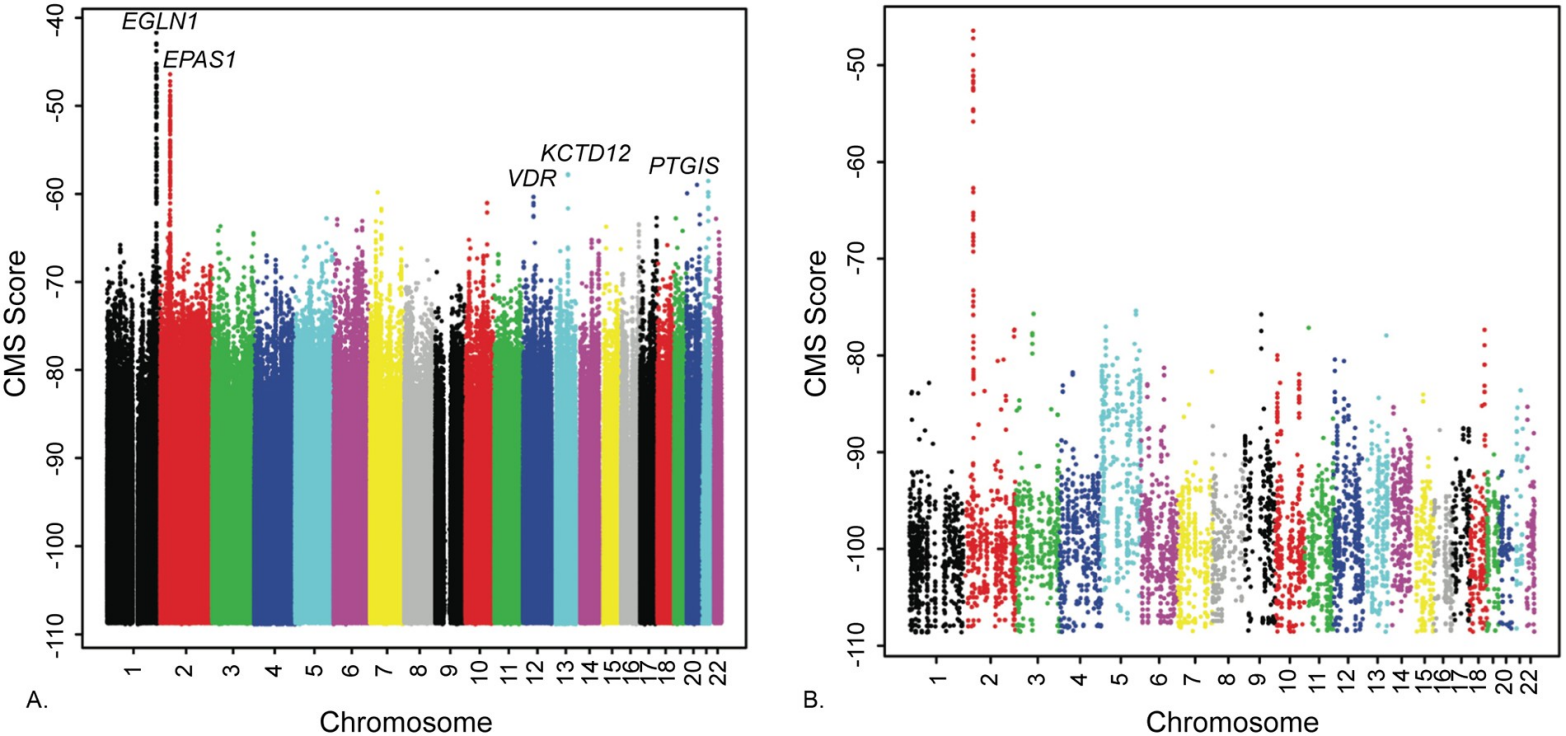
Manhattan plot of the CMS score across the autosome. The x-axis represents the chromosome number and each dot represents one SNV. A) All autosomal SNVs; B) SNVs that are present in the high-coverage reference Denisovan genome and Tibetans but uncommon (MAF<5%) in Yoruba, Europeans, Native Americans and Asians. CMS scores all have negative values, with higher scores corresponding to stronger positive selection signals.

We used the Composite of Multiple Signals (CMS) test[28] to map regions under positive selection in the Tibetan genome (Fig 3). At each genomic variant, CMS evaluates the likelihood of five test statistics (iHS, XP-EHH, ΔiHH, F_ST_ and ΔDAF) under two models: a null model that assumes neutral evolution and an alternative model that incorporates a variety of scenarios involving recent strong positive selection (see S1 Supplementary Methods). CMS then combines information from the five test statistics in a Bayesian framework to create an aggregated CMS score. CMS and its components (iHS, F_ST_ and XP-EHH) have been successfully applied to identify adaptive sweeps originating both from the same population[12,28,29] and from archaic introgression[30–32].

We first calculated the CMS score at each SNV using the 27 Tibetan genomes, with 62 Han Chinese genomes from the 1000 Genomes (1KG) Project[18,33] included as a comparison population (Supplementary Methods). In order to evaluate the statistical significance of our top candidates, we performed coalescence simulations with cosi[34] under a neutral model, using the demographic model that we estimated with ∂a∂i[27] (Supplementary methods). Comparing the CMS scores from our observed data and the null distribution from coalescence simulations, we identified 377 candidate SNVs with FDR < 0.3; 100 of these SNVs are within 350kb of *EGLN1* and 199 are within 350kb of *EPAS1*. We listed all the SNVs with FDR<0.3 in S2 Table.

**Fig 4:**
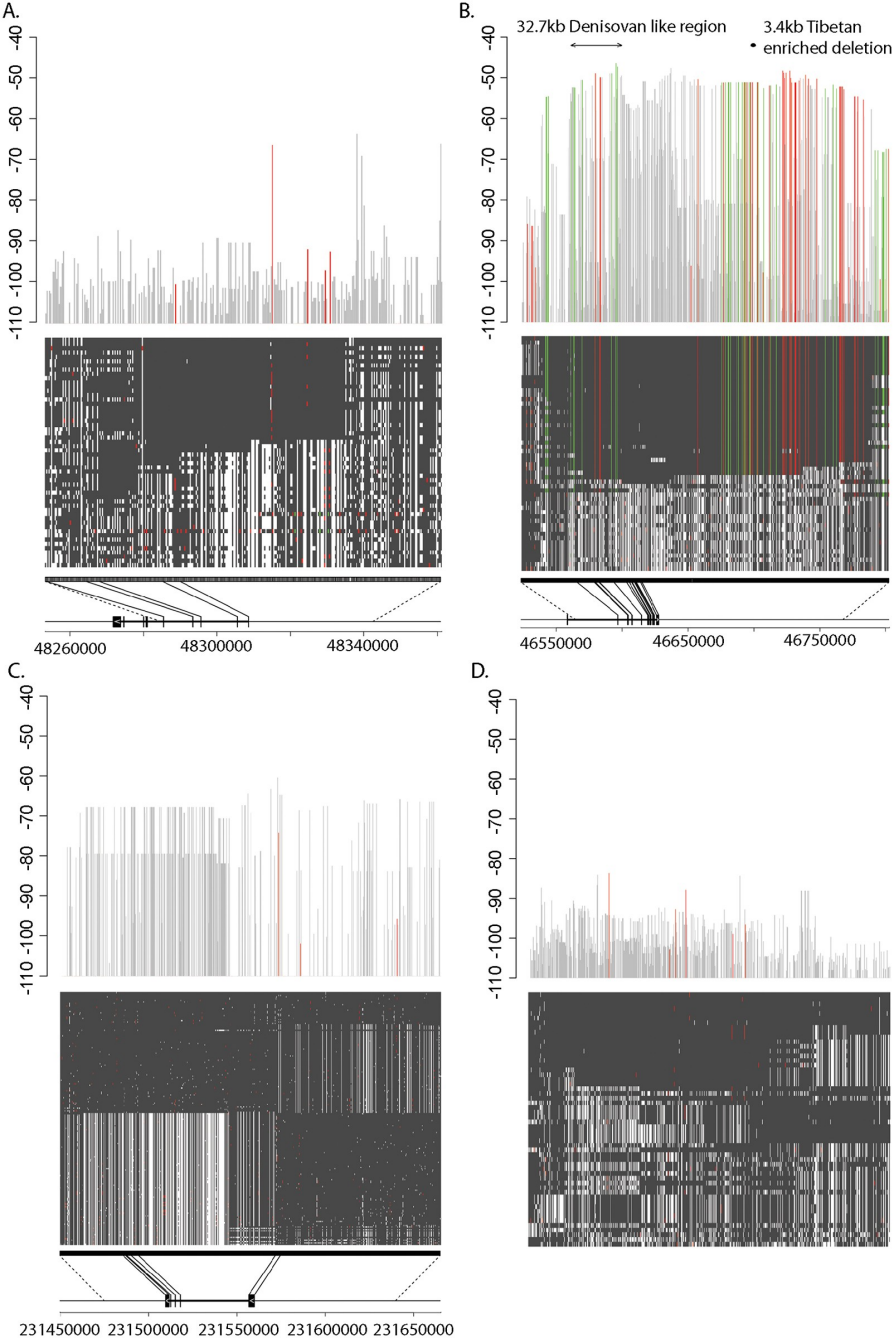
Haplotype map and CMS score in four genomic regions. The upper bar plot demonstrates the CMS values at each SNV. The middle plot shows the haplotype structure of 27 Tibetan genomes in the region; each row represents one genome and each column is one SNV aligned with its CMS score in the upper figure. Both red and green color indicates uncommon variants (MAF<5%) in Yoruba, Europeans, Native Americans and Asians; variants in green in addition must be in the high-coverage reference Denisovan genome. When applicable, the lower plot represents the gene model for the protein-coding gene in the region, with each vertical line representing one exon. a) *VDR* gene region (Chr12:48257328–48357328); b) *EPAS1* gene region (Chr2: 46533376–46792633). The arrowed block above the bar plot indicates a previously identified 32.7kb region enriched for Denisovan variants; the dot above the bar plot indicates a previously identified deletion common in Tibetans (chr2: 46694276–46697683); c) *EGLN1* gene region (Chr1: 231457651–231657496); d) a 200-kb genomic region that presumably underwent no positive selection (showing Chr21: 32800690–33000356, the 200-kb region with the median CMS score).

Because CMS scores between nearby SNVs are often correlated due to linkage disequilibrium, we also performed a region-based CMS test by dividing the genome into 200kb consecutive regions[12], with the test statistic equal to the highest CMS score within each region. Table 2 lists the top 10 regions with the highest CMS scores. The top regions are in the proximity of *EPAS1* and *EGLN1* genes, which were previously reported to be responsible for high-altitude adaptation among Tibetans[12,13]. One of the regions overlaps the gene *VDR*, which encodes the vitamin D3 receptor. Mutations in this gene can cause an abnormality in vitamin D metabolism, which may in turn lead to rickets[35] and preterm birth[36]. We examined the evidence of positive selection on the three SNVs with the best-CMS scores in this 200kb region. Based on the Complete Genomics (CG) 1000 Genome panel data[18,33], the alternative alleles of top 2 SNVs (Chr12:48363253 G->C and Chr12:48359527) are infrequent in Han Chinese (MAFs: 1.6% and 3.2%), but the MAFs were comparable between Tibetans (31.5% and 31.5%) and Europeans (32.1% and 31.4%). The third ranking SNV (Chr12: 48337328 C->T) has a minor allele frequency of 2.4% in Han Chinese, 0.0% in Europeans, 17.0% in Yoruba and 25.9% in Tibetans. The iHS value at this location is 4.22 standard deviations higher than the genome-wide mean, which is about 13,000 times more likely under the alternative model than the null. Fig 4a displays the haplotype map in Tibetans surrounding this region. The focal SNV is 394 bp upstream of the first exon of the transcript AK309587, overlapping the binding site of three transcription factors: *TCF7L2*, *MAFK* and *MAFF*[37]. Both a DNaseI hypersensitivity assay and H3K27me3 histone marker signature in the K562 cell line indicates this is a potential noncoding regulatory region.

**Table 2:** Top 10 regions with the highest CMS scores (hg19 coordinates), with connecting 200-kb windows merged.

Chr	Start	End	Highest CMS score	Genes in the region
1	231200001	232000000	-41.7	*EGLN1*, *SPRTN*, *TSNAX*, *SNRPD2P2*, *EXOC8*, *DISC1*, *DISC2*, *TSNAX-DISC1*, *GNPAT*, *C1orf131*, *TRIM67*
2	46400001	46800000	-46.4	*EPAS1*, *RHOQ*, *PRKCE*, *ATP6V1E2*
13	77200001	77600000	-57.8	*KCTD12*,*BTF3P11*,*CLN5*,*FBXL3*
21	37800001	38000000	-58.5	*CLDN14*
20	48000001	48200000	-59.0	*KCNB1*,*PTGIS*
7	35400001	35600000	-59.8	None
20	1800001	2000000	-59.9	*PDYN*,*SIRPA*
12	48200001	48400000	-60.3	*VDR*,*COL2A1*,*HDAC7*,*TMEM106C*
10	101600001	101800000	-61.0	*ABCC2*,*DNMBP-AS1*,*DNMBP*
7	52400001	52800000	-61.7	None

Another top 10 ranking region overlapped the *KCTD12* gene (potassium channel tetramerization domain containing 12), a component of the G-protein-coupled receptors for γ-aminobutyric acid (GABA) receptor[38]. This region contains 3 SNVs with FDR < 0.05 from our single-variant CMS test. The strongest signal (Chr13: 77399462) among these three SNVs has an observed minor allele frequency of 18.5% in Tibetans, 0.06% in dbSNP (build 142) and 0% among Yoruba, Europeans, and Han Chinese in the CG 1000 Genomes panel. The normalized integrated haplotype score (iHS) value at this location was 4.90 standard deviations higher than the genome-wide mean, which is ~48,000 times more likely to occur under the alternative model than under the null model. This indicates that the haplotype homozygosity around the derived allele is much higher than around the ancestral allele, a scenario consistent with strong positive selection. The three SNVs are located in genomic regions with potential regulatory function. Chr13: 77399462 T->C and Chr13:77398842 G->A overlap with histone mark H3K27Ac annotation and are within enhancer regions, while Chr13:77405193 G->A is at a DNase I hypersensitive site and has a strong signature of H3K27me3 in the K562 (myelogenous leukemia) cell line[37,39].

We next examined Gene Ontology (GO)[40] annotations associated with genes in the top 0.2% of 200kb windows. We evaluated the statistical significance for over-representation of any GO term by repeated sampling without replacement from all 200kb regions in our dataset (Table 3). The following GO terms related to hypoxia responses were over-represented: GO:0071456 (cellular response to hypoxia), GO:0036294 (cellular response to decreased oxygen levels), and GO:0071453(cellular response to oxygen levels). In addition to two genomic regions surrounding *EPAS1* and *EGLN1*, we identified one additional gene related to hypoxia response in GO:0071456: prostaglandin I2 synthase *(PTGIS)*. *PTGIS* converts prostaglandin H2 to prostacyclin, an effective vasodilator and inhibitor of platelet activation[41]. The SNV with the highest CMS score in this region (Chr20: 48175598 C->T) has a normalized XP-EHH value of 3.67, which was 51 times more likely under the alternative model than the null. The derived allele frequency was 85.2% in Tibetan, 42.7% in Han Chinese, 32.1% in Europeans, and 8.9% in Yoruba. The SNV is in the first intron of *PTGIS*, overlapping DNase I hypersensitivity regions, potential *ZNF217* transcription factor binding sites and H3K36me3 histone mark signatures in the NT2-D1 (pluripotent embryonic carcinoma) cell line[37].

**Table 3:** Over-represented GO terms within the top 0.2% CMS windows with q-value <0.05.

GO term	description	q-value	Genes
GO:0050892	intestinal absorption	*0*.*0295875*	*VDR*, *ABCG8*, *ABCG5*
GO:0071456	cellular response to hypoxia	0.0295875	*PTGIS*, *EPAS1*, *EGLN1*, *PRKCE*
GO:0036294	cellular response to decreased oxygen levels	0.0295875	*PTGIS*, *EPAS1*, *EGLN1*, *PRKCE*
GO:0071453	cellular response to oxygen levels	0.0295875	*PTGIS*, *EPAS1*, *EGLN1*, *PRKCE*

Because of the lower variant-calling qualities at genomic insertions and deletions (indels), we could not obtain reliable results from haplotype-based tests (XP-EHH, iHS and ΔiHH) on indels. Instead, we used the Population Branch Statistic (PBS)[13] to search for signatures of positive selection. To calculate the significance levels of the PBS scores, we estimated the null distribution on the same simulation dataset as in the CMS test. We found that indels with the best PBS signals tended to fall into regions having the highest CMS scores. Specifically, 6 out of the top 10 indels are also within the top 10 200kb regions with the highest CMS scores, all in the *EPAS1* and *EGLN1* regions (S3 Table; p = 1.1x10^-15^).

### Mapping of variants under positive selection in the *EPAS1* and *EGLN1* regions

We fine-mapped SNVs under positive selection in the *EPAS1* region by combining the CMS scores with the population allele frequencies in major continental population groups (Europeans, Han, Native Americans and Yoruba) (Fig 4b). We discovered 199 SNVs with FDR < 0.3 from the *CMS* test (*CMS*_*q<0*.*3*_) in the *EPAS1* region (S4 Table), which spans 321 kb from the first intron of *EPAS1* to 257 kb downstream of the last exon of *EPAS1*. Of these 199 SNVs, 64 were in the intronic region and the remaining 135 were in the 3’ downstream region. These SNVs are enriched for alleles present in the Denisovan genome and rare in non-Tibetan populations (MAF<0.05 among Yoruba, Europeans, Native Americans and Asians). Specifically, 16.6% of *EPAS1 CMS*_*q<0*.*3*_ SNVs meet this criterion, as compared to 0.3% genome-wide (p<2x10^-16^).

A previous study identified a 32.7kb region (Chr2: 46567916–46600661) in *EPAS1* that was highly differentiated between Han and Tibetans and also has a high proportion of Denisovan-like variants[16]. In our CMS test result, 2 out of the top 3 SNVs with the highest CMS score in the *EPAS1* region (46597756 and 46598025 on Chromosome 2) were in this 32.7kb region. After excluding rare variants (SNVs with MAF <0.05 in both Han and Tibetans), the average F_ST_ between Han and Tibetans in this 32.7kb region was 0.28. However, the haplotype extends far beyond the previously reported 32.7 kb region and contains genomic regions at least 150 kb downstream of *EPAS1*. Fig 4b illustrates the Tibetan haplotypes in *EPAS1* and its 3’ region. The linkage disequilibrium between SNVs (both Denisovan-like and not) was high among Tibetans (S5 Table), suggesting a slow decay of haplotype homozygosity. The small indel with the highest PBS score was a 4bp deletion located in the 2nd intron of *EPAS1* (Chr2:46577800–46577803). Three out of the four nucleotides affected by this deletion are highly conserved in mammals. This indel overlaps an activating H3K27Ac mark in seven cell lines and the binding sites of three transcription factors: the polymerase subunit *POLR2A*, the HIF co-activator *EP300*[42], and *GATA2*, which is key in the control of erythroid differentiation[43]. The observed frequency of the derived allele (deletion) was 62.9% in Tibetans, 0.8% in Han Chinese and 0% in Europeans, corresponding to a PBS score of 0.600, which was ranked 3^rd^ among all the genomic variant (indels and SNVs) in the *EPAS1* gene.

In addition to the 32.7 kb block, we also identified a second 39.0kb candidate block downstream of *EPAS1* coding region on the haplotype (Chr2: 46675505–46714553); this block was highly differentiated between Han and Tibetans and enriched for Denisovan variants (S3 Fig). Within this block, 88.2% (60 out of 68) of common Tibetan SNVs (derived allele frequency >50%) are present in the Denisovan genome, as compared to 81.4% (48 out of 59) in the previously reported 32.7kb region. The proportion of SNVs shared with the Neanderthal genome is 26.5% (18 out of 68) for the new 39.0kb block as compared to 25.4% (15 out of 59) for the previous 32.7kb block. In this block, the average F_ST_ between Han and Tibetans for SNVs common in at least one of the two populations is 0.304 (as compared to 0.279 for the previously reported region). Intriguingly, this region also fully contains a 3.4kb Tibetan-enriched deletion region that showed footprints of positive selection in a previous study[44] (Fig 4b). Among our Tibetan samples, this deletion has an allele frequency of 59.2% and is in high LD (r^2^ = 0.86) with the SNV with the highest CMS in the *EPAS1* region (Chr2: 46597756; S6 Table).

We estimated the origin age of the selective sweep on the adaptive *EPAS1* haplotype to be 12 kya (95% CI: 7 kya to 28 kya) using a maximum likelihood approach based on coalescence simulations of the demographic model described in Table 1 (see S1 Supplementary Methods for details). Our estimate is consistent with a previous estimate of 12 kya to 15 kya[44]. Using this point estimate of selection start time and assuming that the adaptive haplotype indeed originated from Denisovans or a closely related population, we further inferred that the introgression of this haplotype most likely occurred between 32 and 12 kya. We also estimated the gene-tree divergence time between the adaptive haplotype in Tibetans and the available Denisovan DNA sequence to be 997 kya (95% CI: [868, 1,139]). From the gene-tree divergence and calibrated models of Denisovan demographic history[45], we estimated a population divergence time of 868 kya (95%CI: 952 kya to 238 kya) between the Densivan population and the archaic population that contributed the adaptive *EPAS1* haplotype.

Previously, we identified two non-synonymous variants in the *EGLN1* gene (c.12C>G, p.D4E; c.380G>C, p.C127S) which together act as a co-adapted gene complex contributing to high-altitude adaptation in Tibetans[14]. In the current dataset, the c.12C>G variant was absent from any other populations in the 1KG dataset and had high F_ST_ (0.70) and ΔDAF (0.70) values, indicative of strong positive selection. The c.380G>C variant has a derived allele frequency of 77.8% (95% CI: [66.7%, 88.9%]) in Tibetans and 44.9% (95% CI: [40.0%, 50.0%]) in Han Chinese, consistent with previous findings[14]. The CMS score is -60.43 for c.12C>G and -72.08 for c.380G>C, both ranked among the top 0.01% variants genome-wide. We also identified a novel rare *EGLN1* variant (c.C358T, p.P120L) within one Tibetan individual, who also carries the c.380G>C but not c.12C>G variant. In our dataset this variant has a frequency of 1.85% (95% CI: [0.05%, 9.89%]) in Tibetans, 0.81% (95% CI: [0.02%, 4.41%]) in Han Chinese and is absent from other populations in the 1KG dataset.

### Mapping regions with high Denisovan ancestry in the Tibetan genome

To evaluate whether any genomic regions with Denisovan introgression other than *EPAS1* may contribute to Tibetan high-altitude adaptation, we first calculated D-statistics[46] to evaluate the overall level of Denisovan admixture in the Tibetan genome. When comparing Tibetans to Yoruba, we observed a D of 0.011 (95% CI: 0.0068 to 0.0163), indicating a significant excess of Denisovan admixture among Tibetans. However, we observed no significant difference in D between Tibetans and Han Chinese (D = -0.0007, 95% CI -0.0027 to 0.0021), demonstrating that the genome-wide level of Denisovan admixture does not differ substantially between Tibetans and Han Chinese, and is consistent with a previous report[47]. Using the Q statistic of Rogers and Bohlender[48], we estimated that the proportion of Denisovan admixture in Tibetans is 0.4% (95% CI: 0.2% to 0.6%, see Methods and S4 Fig). Briefly, Q is an f_3_ ratio estimator of admixture[48]. Given a sample from three populations X, Y and N, so that X and Y are more recently diverged from each other than either is from N, the calculation of Q uses a ratio of expectations to calculate the total admixture from N into Y, in a similar way as other f_3_-ratio estimators[46]. Q has the advantage of requiring only a single archaic individual, and uses external information about ghost admixture to correct bias due to admixture from an unsampled archaic population (Neanderthal).

To investigate the variation in Denisovan admixture across the genome among Tibetans, we first used an LD-based statistic S*[32] to identify introgressed regions originated from Denisovans. S* searches for haplotypes that share a substantial amount of similarity with the archaic genome and are present in the population of interest but not in the comparison population (see S1 Supplementary Methods). In our study, we used Han Chinese as the comparison population and Denisovan alleles as the archaic genome to search for Denisovan haplotypes absent among the Han Chinese genomes and present in at least one Tibetan genome (Supplementary Methods). In total, S* identified 660 50kb candidate regions using this procedure (S5 Fig; S7 Table). As an alternative approach, we also developed a new statistic, D*, which normalizes the D-statistics to enable the identification of admixed regions with calibrated Type I error (Methods). We used D* to compare Tibetans to Han Chinese for all 200kb genomic regions (S5 Fig). With this procedure, we identified 11 regions with significant proportions of Denisovan admixture after multiple-testing correction (FDR < 0.30). The region containing *EPAS1* ranked second, with D* = 6.2 (p = 6.1 x 10^−6^). Six of 11 regions identified by D* overlapped with the 660 regions identified by S*, including the region containing *EPAS1* (Table 4).

**Table 4:** 200-kb genome regions with higher Denisovan ancestry in Tibetan than in Han Chinese in both S* and D* tests.

Chr	start	end	raw D-value	D*	p-value	q-value	Genes in the regions
7	26800001	27000000	0.86	6.25	6.1E-06	2.7E-02	*SKAP2*
2	46400001	46600000	0.56	6.15	6.1E-06	2.7E-02	*PRKCE*,*EPAS1*
2	47600001	47800000	0.65	5.92	6.1E-06	2.7E-02	*MIR559*,*MSH2*,*EPCAM*,*KCNK12*
5	200001	400000	0.52	4.99	6.1E-05	1.4E-01	*CCDC127*,*PDCD6*,*SDHA*
12	8800001	9000000	0.63	4.63	1.2E-04	1.7E-01	*MFAP5*,*RIMKLB*,*A2ML1*
4	100400001	100600000	0.48	4.58	1.2E-04	1.7E-01	*TRMT10A*,*C4orf17*,*MTTP*

Next, to test for evidence of adaptive Denisovan introgression, we compared the location of the Denisovan DNA regions identified by either S* or D* to the regions with the highest CMS scores. The top candidate identified by this analysis is *EPAS1*. Since we were interested in identifying novel adaptive Denisovan introgression, we removed the *EPAS1* region and evaluated the statistical significance of the observed number of overlaps. To calculate the p-value, we permuted the CMS scores of all 200kb genomic regions to generate the distribution of overlaps under the null hypothesis that no additional Denisovan-like DNA was positively selected in Tibetans. When we compared the Denisovan-like genomic regions to the top 0.2% of CMS regions, we found no overlapping region with D* and only one with S* (Chr2:42200001–42400000). In the top 1% of CMS regions, we identified no overlapping regions with D* and three overlapping regions (Chr2: 42200001–42400000; Chr1: 79200001–79400000; Chr1:1200001–1400000) with S*; however, the p-values from all tests were non-significant. Therefore, we find no evidence that additional Denisovan-like DNA has contributed to Tibetan high-altitude adaptation. We summarized the CMS scores of Denisovan variants that are present in Tibetans but uncommon among major population groups in Fig 3b.

## Discussion

Our demographic history estimates from ∂a∂i suggest that population structure existed between the Tibetan and Han ancestral subpopulations as early as 44 to 58 kya, with high rates of admixture maintained between the two subpopulations until around 9 kya. This agrees well with the findings of Lu et al.[49] that the early colonization of the Tibetan plateau occurred between 62kya and 38kya, and the post-Last Glacial Maximum (LGM) arrival at the Tibetan plateau could be between 15kya and 9kya. Moreover, archaeological evidence [1,50] suggests that the Tibetan plateau was occupied during the Late Pleistocene, roughly coinciding with the time of Han-Tibetan separation in our demographic model. Interestingly, MSMC did not detect a decrease in relative cross coalescence rate between Han and Tibetans until 3 to 9 kya. We hypothesized that this discrepancy was due to an inability of MSMC to differentiate between panmixia and high rates of gene flow between Tibetan and Han Chinese populations between 9kya and 54kya. To test this hypothesis, we conducted coalescent simulations to evaluate the ability of MSMC to accurately estimate the Tibetan-Han divergence time under a model where the two populations diverged at 54 kya but experienced a large amount of gene-flow (6.8x10^-4^ and 9.0x10^-4^ per generation per chromosome) until 9 kya. As shown in S6 Fig, MSMC was indeed unable to detect the ancestral population split at 54 kya. The observed pattern of estimated relative cross-coalescence rate was consistent between the observed data and simulations based on the best-fitting ∂a∂i model, with no decrease in the relative cross-coalescence rate prior to 10 kya. Conversely, when we conducted coalescence simulation using the simplified demographic model predicted by MSMC (i.e., Han and Tibetan separated 7,000 years ago) and ran ∂a∂i, we found that ∂a∂i accurately recovered the simulated divergence date (S8 Table), providing support for the ∂a∂i model with an earlier divergence date but a high migration rate between the two populations until approximately 9 kya.

Our admixture analysis in the *EPAS1* region confirmed findings from previous studies that a common Tibetan haplotype in this region contains excessive Denisovan-like DNA[16], but also showed that on a genome-wide level, the amount of Denisovan admixture in Tibetans (0.4%) is similar to that of Han Chinese. This suggests that at least one major introgression occurred from the archaic population to the common ancestor of Han and Tibetans. The high-altitude adaptive haplotype in the *EPAS1* region may have been acquired prior to Han-Tibetan divergence, but was either lost or present at low frequencies in Han due to the lack of selection. This explanation agreed with the hypothesis proposed by Huerta-Sanchez et al[16], who posit that the *EPAS1* haplotype may have been introduced prior to the separation of Han and Tibetans, considering the presence of the Tibetan *EPAS1* haplotype in a single Han Chinese individual. Alternatively, it is possible that the low frequency of the Tibetan *EPAS1* haplotype in China is the result of relatively recent migration from Tibet[16] and that the haplotype was originally introduced into Tibet after the Han-Tibetan divergence. This alternative explanation is supported by our 95% C.I. for the introduction of the *EPAS1* haplotype into the ancestral Tibetan population (32 to 12 kya) which is later than the previous estimates for the date of Denisovan admixture into modern humans (44.0 to 54.0 kya)[51]. Other than the *EPAS1* haplotype, we observed no evidence of other Denisovan-like DNA segments contributing to high-altitude adaptation in Tibetans, given that we did not detect significant overlaps between introgressed regions and the top 1% of CMS regions. However, we cannot rule out the possibility that with larger sample sizes, subtle adaptive introgression signals may be detected.

In addition to the previously reported 32.7kb haplotype block in *EPAS1*[16], we identified a much larger haplotype that contains both the *EPAS1* genic region and at least 150kb 3’ of *EPAS1*. However, the extensive LD within the *EPAS1* and its 3’ region prevented us from pinpointing the location of the adaptive mutation. Interestingly, the selected haplotype (Fig 4b; S3 Fig) contains many Denisovan-like and non-Denisovan-like variants, and many top candidate SNVs in our analysis, as well as the previously reported 3.4kb Tibetan enriched deletion, were absent from the high-coverage Denisovan genome. This observation is reflected in the estimated population divergence time of 952 to 238 kya between the population represented by the Denisovan reference genome and the archaic population that admixed with Tibetans (Supplementary Methods). This estimate is broadly consistent with previous evidence that the archaic Denisovan-like population that admixed with modern human populations separated from the population represented by the Denisovan reference genome between 276 kya and 403 kya[52]. Thus, the advantageous *EPAS1* haplotype shows far greater similarity to Denisovans than would be expected in the absence of archaic admixture, but substantial mismatch should be expected given the apparent diversity of archaic populations outside of Africa[53].

Our CMS test combines five statistical tests of positive selection (iHS, XP-EHH, F_ST_, ΔiHH and ΔDAF), offering a more robust performance across a wide variety of scenarios compared to any single test[28]; this allowed us to identify novel candidate genes contributing to adaptations on the Tibetan plateau. One of the candidate gene for adaptive selection is *VDR*, a gene encoding nuclear hormone receptor for 1, 25 dihydroxyvitamin D_3_ and functions in Vitamin D metabolism. Vitamin D is a secosteroid nutrient that plays an important role in calcium homeostasis and bone mineralization, and mainly comes from two sources: 1) through skin exposure to sunlight and 2) food, such as fish, milk and egg yolk. Deficiency in vitamin D leads to impairment of bone mineralization and skeletal deformities, known as rickets in children and osteomalacia and osteoporosis in adults [54]. Low level of vitamin D has also been associated with cancers, diabetes, autoimmune diseases, hypertension, and infectious disease[35,55]. In a study involving vitamin D status in a cohort of 63 Tibetans [54], the proportion of nomadic Tibetans with vitamin D deficiency (25(OH)D <75 nmol/L) was 100% with 80% of people having severe deficiency (25(OH)D < 30nmol/L); the proportion of non-nomadic Tibetans with vitamin D deficiency ranges from 40% to 83%. Consistently, in another study, 61% of Tibetan children suffer from rickets and 51% have stunted growth[56]. Such a high prevalence of vitamin D deficiency may be explained by the traditional Tibetan diet consisting of barley, yak meat and butter tea which are poor sources of vitamin D, and clothing habits in cold temperatures which allow for minimal skin exposure to the sunlight [54]. Therefore, we hypothesize that *VDR* gene is positively selected to compensate for the lack of vitamin D, the mechanism of which remains to be determined. Previously, it has been shown that genomic regions bound by *VDR* were under adaptive selection in the human genome[57].

A second promising candidate identified in our CMS test is *PTGIS*, a gene associated with three hypoxia-response GO terms (0071456, 0036294,0071453) and regulated by hypoxia-inducible factor 1 (HIF-1). *PTGIS* encodes prostaglandin I2 synthase, which participates in the synthesis of prostanoid. Previous studies have shown that the expression of *PTGIS* is induced by hypoxic conditions in human lung fibroblast cells and cancer cell lines[58], and can activate vascular endothelial growth factor. Therefore, it is possible that a Tibetan-unique genomic variant may induce vasodilation and angiogenesis in response to hypoxia by altering the expression of *PTGIS*.

The CMS test also identified *KCTD12* as a new high-altitude adaptation candidate. Previously, it has been shown in B16 murine melanoma cells that *KCTD12* is up-regulated in hypoxic conditions[59], and thus it may play a role in the hypoxic responses. This gene is predominantly expressed in fetal organs, suggesting a potentially important role in early development, but dramatically lower levels in adult tissue including brain and lung[60]. *KCTD12* is differentially expressed in human pulmonary endothelial cells upon 48 hours of hypoxia exposure[59] and noted as one of 40 CpG sites with the greatest difference in methylation levels between highland and lowland Oromo Ethiopians[61].

Our functional annotation of Tibetan genomic variants identified a frequent protein-coding SNV in *ALPP*, a gene associated with pregnancy losses[25]. Since Tibetans tend to have higher birth weight in high altitude environments compared to lowland native populations living at similar altitudes[62], it is possible that the nonsynonymous variant in *ALPP* may affect the birth weight, development, and pregnancy outcomes in Tibetans. For every 1000 meters of altitude, birth weight decreases an average of 100 grams due to restricted fetal growth[63–66], with a three-fold increase in the number of infants born small for gestational age (SGA) at high altitude[67]. Weights of infants born at high altitudes to mothers of highland origin, however, are greater than those of lowland origin. This is shown specifically in infants born to Bolivian versus European women in the Andes, whose birth weights are about 300g higher in the former group, while no difference is reported by ethnicity at low altitude[67]. Whether these putatively adaptive markers play roles in this process remains to be determined.

**Fig 5:**
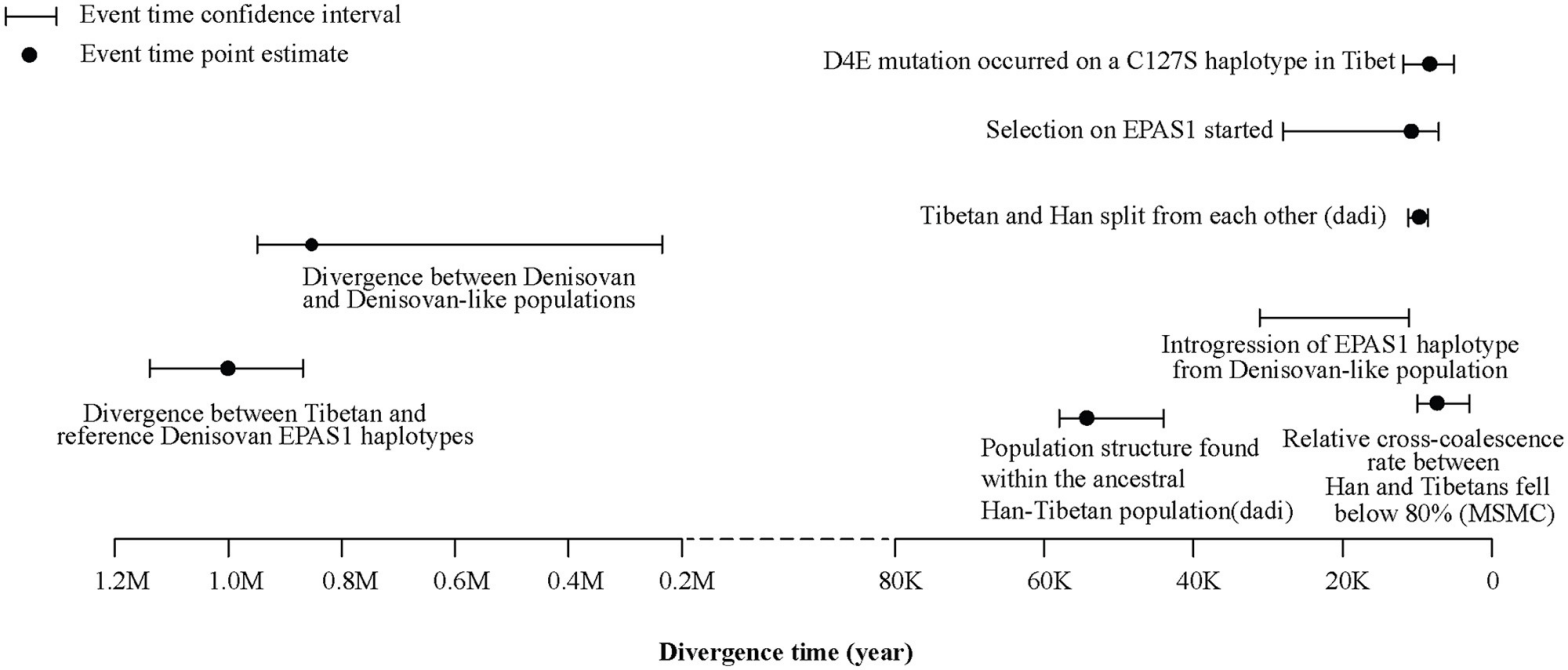
Timeline of important evolutionary events in the demographic history of Tibetans. The horizontal axis represents the estimated time of events (in years) in the past. The age of the *EGLN1* D4E mutation is based on previous estimates.

In summary, we performed a comprehensive genomic analysis on whole-genome sequence data from 27 Tibetan individuals. Our analyses detected evidence of population structure between the ancestral Han and Tibetan subpopulations beginning between 44 and 58 kya, although admixture rates between the two subpopulations remained high until around 9 kya. The Denisovan *EPAS1* haplotype introgressed into the Tibetan population between 12 and 32 kya, and positive adaptive pressure on this haplotype began between 7 and 28 kya. We summarized the dates of important demographic events in Fig 5. The Our CMS test identified novel candidate genes for high-altitude adaptation including *KCTD12*, *VDR and PTGIS*, and also generated a list of candidate variants within the *EPAS1* gene region. We estimated that 0.4% of the Tibetan genome are introgressed DNA from Denisovans, although *EPAS1* is probably the only introgressed locus that was influenced by strong positive selection in Tibetans. Our study provided a rich genomic resource of the Tibetan population and generated hypotheses for future positive selection tests.

## Materials and methods

### Sequencing samples and variants calling

Our study subjects included 27 Tibetan individuals recruited from Tibetans living in San Diego, California (n = 3), Salt Lake City, Utah (n = 5), and the United Kingdom (n = 19). The sample type and place of origin information are listed in S9 Table. Whole-genome sequencing and variant-calling was performed by CG (v2.0 for the first 17 samples and v2.5 for the remaining 10 samples). Variants with genotyping quality less than 30 (i.e., more than 1 calling error among 1000 variants) were converted to missing genotype calls, and sites with more than 5% missing genotype rate were removed. To identify additional variants enriched for higher genotyping error rates, we compared 62 genomes that had been sequenced in both CG public genome data[33,68] and 1KG Project[18] Phase I data. We identified 652,899 SNVs with more than 5% discordant genotype calls between these two datasets, and removed them from all further analyses. We then performed haplotype phasing and imputation using Shapeit2[69]. After previous steps, 10,405,415 SNVs remained for subsequent analyses.

### Whole-genome positive selection scan using Composite of Multiple Signals (CMS) test

The CMS test combines the signals from five different tests (ΔiHH, iHS, XP-EHH, F_ST_ and ΔDAF) to create a single test statistic[28]. On each variant, CMS calculates the posterior probability of the variant being selected in a naïve Bayes framework by taking the product of the posterior probabilities from each of the five tests. A higher CMS score is consistent with a stronger signal of positive selection. Our implementation of the CMS test closely follows the recommendations given in the previous report[28]. Briefly, we first performed coalescence simulations using cosi[34] based on the demographic model estimated by ∂a∂i. To simulate genetic data under various scenarios of selective sweeps, we used the following selection coefficients (*s*): 0.02, 0.03 and 0.04; the following start times of selection: 0, 100, 200, 300 and 400 generations ago (the last one represents the oldest selection start time that cosi may simulate within our model); and the following non-reference allele frequencies at the conclusion of the sweep: 0.2, 0.4, 0.6 and 0.8. In addition, we also simulated the scenario where no selective sweep had occurred, which corresponded to the null model in the CMS test. In each situation, we performed 1000 simulations. We calculated the CMS scores for all SNVs that passed our quality-control procedures. To calculate their p-values, we also ran the CMS test over the null model that we simulated above. The p-value for a certain CMS score was calculated as the proportion of SNVs in the null model that has the same or higher CMS scores.

### Testing for Denisovan admixture using the D statistic

The *D* statistic tests whether the amount of archaic admixture in one population exceeds that of another population by examining variant allele frequency spectrum in the case population, control population, an archaic population and an outgroup population[70]. In our study, these were selected as Tibetans, Han Chinese/Yoruba, Denisovan and chimpanzee. Let ${\hat{p}}_{1}^{i}$, ${\hat{p}}_{2}^{i}$, ${\hat{p}}_{3}^{i}$, and ${\hat{p}}_{4}^{i}$, be the allele frequency of the i-th variant in the case, control, archaic and outgroup populations, respectively. *D* is defined as:

$$D(P_{1},P_{2},P_{3},O)=\frac{\sum_{i=1}^{n}\left[(1-{\hat{p}}_{1}^{i}){\hat{p}}_{2}^{i}{\hat{p}}_{3}^{i}(1-{\hat{p}}_{4}^{i})-(1-{\hat{p}}_{2}^{i}){\hat{p}}_{1}^{i}{\hat{p}}_{3}^{i}(1-{\hat{p}}_{4}^{i})\right]}{\sum_{i=1}^{n}\left[(1-{\hat{p}}_{1}^{i}){\hat{p}}_{2}^{i}{\hat{p}}_{3}^{i}(1-{\hat{p}}_{4}^{i})+(1-{\hat{p}}_{2}^{i}){\hat{p}}_{1}^{i}{\hat{p}}_{3}^{i}(1-{\hat{p}}_{4}^{i})\right]}$$ (1)

We estimated the confidence interval of *D* using bootstrap sampling with 200 replicates. Specifically, we divided the whole genome into 1MB blocks, and then randomly sampled with replacement the same number of 1MB blocks as in the actual data, each time calculating a sample *D* statistic. The 2.5% and 97.5% quantiles of the 200 *D* statistics values were used as the two end points of the 95% confidence interval.

### Identifying regions of Denisovan admixture using the D* statistic

We developed the D* statistic as a complement to S* to identify local genomic regions with excess archaic admixture in a population. We first intended to use the D statistic to identify 200kb genomic regions with archaic admixture in the case population, but noticed that the variance of the D-statistic was high if the number of archaic variants in a target region was small. This sensitivity of the D-statistic to regions of low archaic gene flow has been noted previously, and alternatives to the D-statistic have been suggested[71]. To illustrate this sensitivity, consider the extreme scenario where only one archaic variant exists in a 200kb genomic region, and this variant was present in only one case genome and absent from all control genomes. In this situation, D will be the largest possible value Eq (1) despite the fact that the evidence supporting an archaic admixture in this region is poor. This property of D makes it unsuitable for prioritizing candidate introgression regions, since the majority of regions with high D values will be those with few archaic variants in modern human genomes.

Previous work has suggested using direct estimators of the quantity of gene flow as an alternative to a method like D-statistics due to the sensitivity of the statistic to population history and other exogenous parameters[71]. However, D-statistics and the related f-statistics vary only in magnitude as a result of these biases, and will only deviate from zero, in expectation, as a result of gene flow[46]. Normalization of D-statistics preserves their desirable characteristics, while eliminating a source of bias.

To normalize D-statistics, we calculated the following statistic (U) for each 200kb genomic region:

$$U(P_{1},P_{2},P_{3},O)=\sum_{i=1}^{n}[(1-{\hat{p}}_{1}^{i}){\hat{p}}_{2}^{i}{\hat{p}}_{3}^{i}(1-{\hat{p}}_{4}^{i})+(1-{\hat{p}}_{2}^{i}){\hat{p}}_{1}^{i}{\hat{p}}_{3}^{i}(1-{\hat{p}}_{4}^{i})]$$ (2)

As can be seen, U is the denominator of the D in Eq (1) and is negatively correlated with the standard deviation associated with D (S7 Fig). To create a normalized D-statistic (D*) that can be used to compare admixture signals across regions, we sort all 200-kb genomics regions according to their U values and then divide the regions into 20 equal-sized groups. Then D* of a 200kb region is equal to its D value divided by the within-group standard deviation. We estimated the significance of a given D* by comparing it to the null distribution of D* generated by coalescence simulation.

### Estimating the proportion of Denisovan admixture

Because only one Denisovan genome is available, we use the statistic Q of Rogers and Bohlender (Equation 11 of [48]), interpreting the primary source of introgression as Denisovan rather than Neanderthal. The expectation of Q depends not only on the fraction, m_D_, of Denisovan admixture but also on m_N_, the fraction of ghost admixture from another archaic such as Neanderthal. Equating observed and expected values defines m_D_ as an implicit function of m_N_. To evaluate this function, we assumed the parameter values in table 3 of [48]. The result is shown as a solid black line in S4 Fig. We repeated this process, swapping the roles of Neanderthal and Denisovan, to estimate m_N_ as a function of m_D_. This result is shown as a solid red line in S4 Fig. The intersection provides a simultaneous estimate of m_D_ and m_N_.

### Availability of data and material

The database of Genotypes and Phenotypes (dbGAP) accession number for the whole-genome sequencing data reported in this paper is phs001338.

### Ethics approval and consent to participate

This study was approved by the Institutional Review Board at University of Utah and at the University of California, San Diego, by the Berkshire Clinical Research Ethics Committee, UK, and by the Western Institutional Review Board (WIRB). Informed consent was obtained from all participants.

## Supporting information

S1 Fig Principal Component Analysis (PCA) on the genomic variants of Tibetans and 5 other populations from 1000 Genomes Project. Each circle in the plot represents one genome. A) The 1^st^ and 2^nd^ principal components; B) The 3^rd^ and 4^th^ principal components; C) the 5^th^ and 6^th^ principal components; D) The 1^st^ and 2^nd^ principal components in the PCA of Chinese and Tibetans only.
(DOCX)

S2 Fig ADMIXTURE analysis on the genomics variants of Yoruba (YRI), Peruvian (PEL), Europeans (CEU), Punjabi (PJL), Chinese (CHS), and Tibetan (TIB) populations. Each column represents one individual and different colors represent different ancestral population; each panel represents one K value. The reported ethnicities were listed on the top of each panel.
(DOCX)

S3 Fig Manhattan plot of the FST score in the EPAS1 region. The x-axis shows the position on chromosome 2, in Hg19 coordinate. The y-axis shows the FST value. Red color indicates Denisovan-like alleles. Only variants with MAF no less than 5% in either Han or Tibetan were shown. A) Chr2: 44000000–49000000. The *EPAS1* region is indicated by the two vertical dotted lines. B) Chr2:46500000–46800000. The two blue vertical dotted lines indicate chr2: 46567916–46600661 (the 32.7kb region reported previously). The two red vertical dotted lines indicate chr2: 46675505–46714553, a second region with high FST and many Denisovan-like variants.
(DOCX)

S4 Fig Simultaneous estimate of Neanderthal admixture (mN) and Denisovan admixture (mD) into Tibetans. Key: solid black, mD given mN; solid red, mN given mD; dashed, 95% confidence regions based on moving-blocks bootstrap; circle, simultaneous estimate.
(DOCX)

S5 Fig Denisovan admixture in Tibetan genomes. The x-axis represents the chromosome number. Each dot represents one 200kb genomic region identified by S*.
(DOCX)

S6 Fig MSMC estimate of relative cross-coalescence rate between Han and Tibetans. The red curve shows the relative cross-coalescence rate based on the whole genome sequencing data; the grey curves show the relative cross-coalescence rate based on 20 coalescence simulations from our best-fitting dadi model with a true Han-Tibetan divergence time of 54 kya but with high rates of gene flow until 9 kya (see Fig 2).
(DOCX)

S7 Fig Standard deviations of D-statistics as a function of U. We sorted all the 200-kb genomic regions by their U values and calculated the standard deviations of the D-statistics within each percentile of U. Each dot represents the D and U for one percentile. The target population is Tibetan and the background population is Yoruba.
(DOCX)

S8 Fig MSMC estimate of relative cross-coalescence rate between 1) Han and European (red) and Han and Tibetan (black). (DOCX)

S9 Fig Site Frequency Spectrum (SFS) comparison between observed data and model prediction. Panel A, B and C corresponds to *∂a∂i* model A, B and C. In each panel, the first two plots show the observed and model predicted SFS heatmaps, respectively; the third plot shows the residual heatmap; the fourth plot shows a histogram of the residuals. Panel D shows the one-dimensional SFS for Han Chinese (left) and Tibetans (right) separately. Within each combination of population and model, the top plot shows the frequencies of variants for each minor allele count, with the red line showing the expected frequencies predicted by the model and blue line showing the observed frequencies; the bottom plot shows the standardized residuals of frequencies within each minor allele count category, assuming the frequencies are Poisson-distributed.
(DOCX)

S10 Fig Distribution of CMS scores either using only 19 simulated Tibetan individuals with no modern admixture (non-admixed), versus using 27 simulated Tibetan individuals with an average of 5.2% modern admixture from Han Chinese (admixed). (DOCX)

S1 Table Nonsynonymous SNVs frequent in Tibetans but not in Yorubans, Han and Europeans. (XLSX)

S2 Table List of all SNVs with FDR<0.3 in the CMS test. (XLSX)

S3 Table Top 10 Small insertion and deletions with the highest PBS scores. The start and end positions are in hg19 coordinates.
(XLSX)

S4 Table SNVs with q<0.3 in CMS test in the EPAS1 region. (XLSX)

S5 Table Linkage disequilibrium (r^2) between the top 30 candidate SNVs in the EPAS1 region. Red-color indicates SNVs present in the Denisovan genome.
(XLSX)

S6 Table LD (r^2) between the 3.4kb deletion and SNVs with q<0.3 in the EPAS1 region. (XLSX)

S7 Table Regions with Denisovan introgression, identified by S*. (XLSX)

S8 Table Dadi's paramter estimate on the Han-Tibetan demographic model predicted by MSMC. We first simulated a 50MB genomic region (with msms) under the MSMC demographic model, and then used dadi to estimate the Han-Tibetan divergence time. The actual demographic parameters are in the Simulated columns, and the dadi estimates are in the Estimated column.
(XLSX)

S9 Table DNA source and place of origin for each Tibetan participant. (XLSX)

S10 Table Parameter bounds for dadi models. (XLSX)

S1 Supplementary Methods Detailed description of methods used in this manuscript (if not covered in the main text). (DOCX)
