# Supplementary material for: Evolutionary history of Tibetans inferred from whole-genome sequencing
Source: PLoS Genet. 2017 Apr 27;13(4):e1006675. doi: 10.1371/journal.pgen.1006675 (PMC5407610; doi:10.1371/journal.pgen.1006675)
Supplement: S1 Fig — Each circle in the plot represents one genome. A) The 1st and 2nd principal components; B) The 3rd and 4th principal components; C) the 5th and 6th principal components; D) The 1st and 2nd principal components in the PCA of Chinese and Tibetans only. (DOCX) [file pgen.1006675.s001.docx]

A.

B.

C.

D.
