# Supplementary material for: Evolutionary history of Tibetans inferred from whole-genome sequencing
Source: PLoS Genet. 2017 Apr 27;13(4):e1006675. doi: 10.1371/journal.pgen.1006675 (PMC5407610; doi:10.1371/journal.pgen.1006675)
Supplement: S2 Fig — Each column represents one individual and different colors represent different ancestral population; each panel represents one K value. The reported ethnicities were listed on the top of each panel. (DOCX) [file pgen.1006675.s002.docx]

A. K=2

B. K=3

C. K=4

D. K=5

E. K=6

F. K=7

G. K=8
