# Supplementary figures and images for: Evolutionary history of Tibetans inferred from whole-genome sequencing
Source: PLoS Genet. 2017 Apr 27;13(4):e1006675. doi: 10.1371/journal.pgen.1006675 (PMC5407610; doi:10.1371/journal.pgen.1006675)

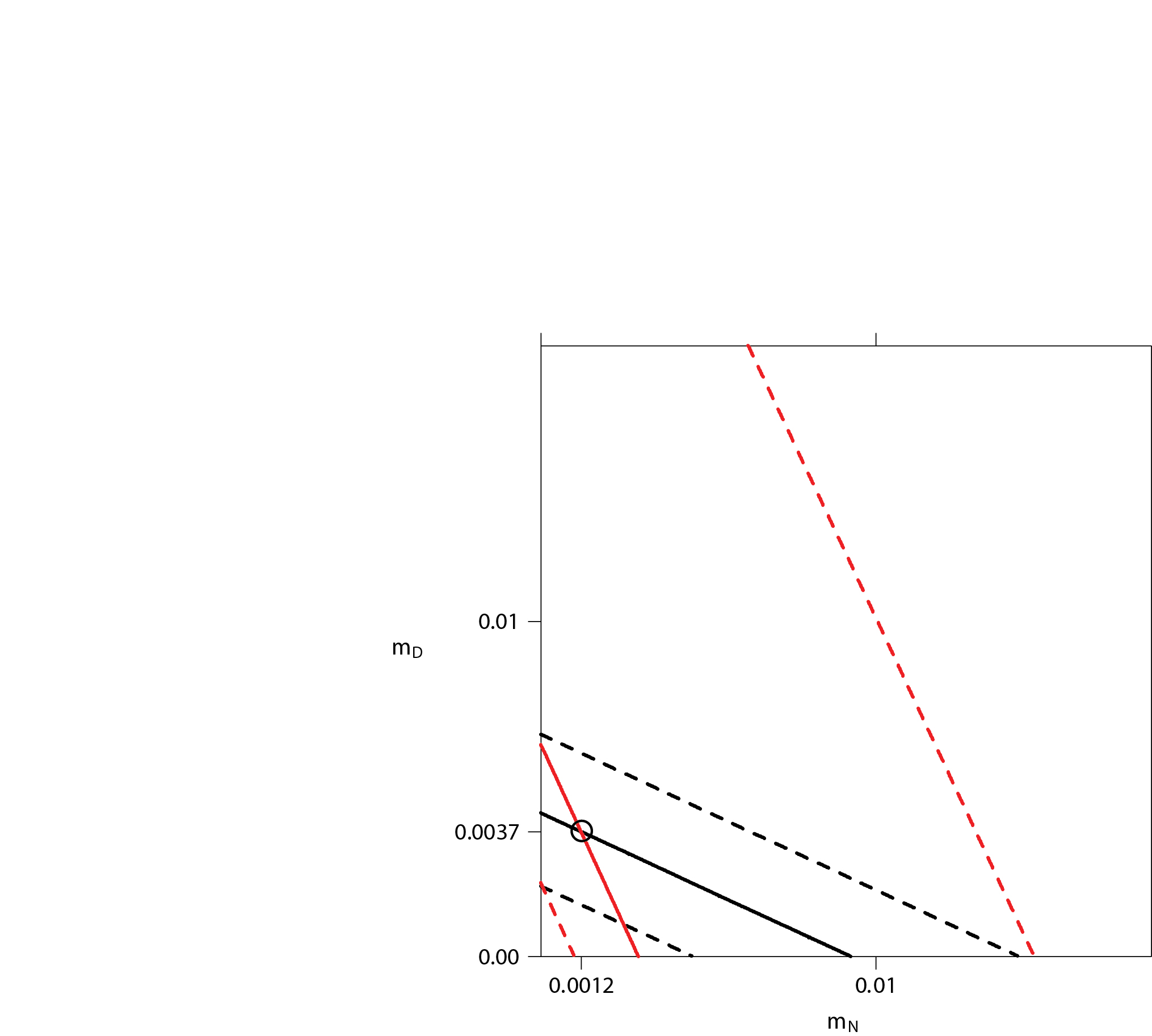

Supplement: S4 Fig — Key: solid black, mD given mN; solid red, mN given mD; dashed, 95% confidence regions based on moving-blocks bootstrap; circle, simultaneous estimate. (DOCX) [file pgen.1006675.s004.docx]

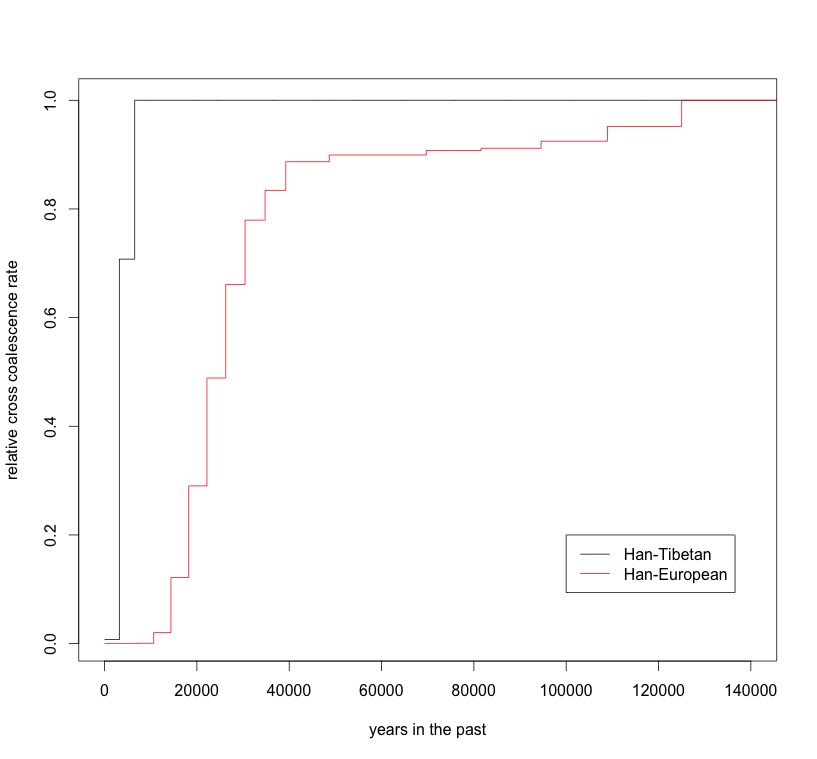

Supplement: S8 Fig — (DOCX) [file pgen.1006675.s008.docx]

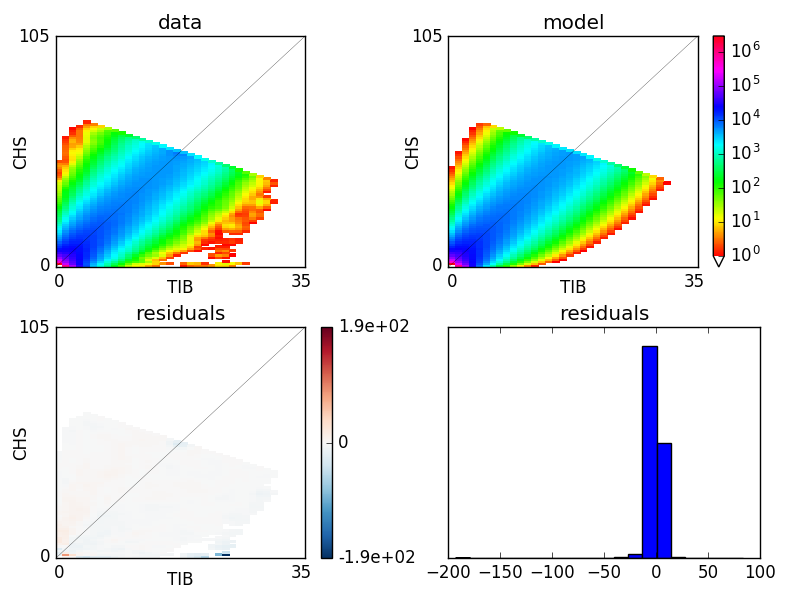


A.


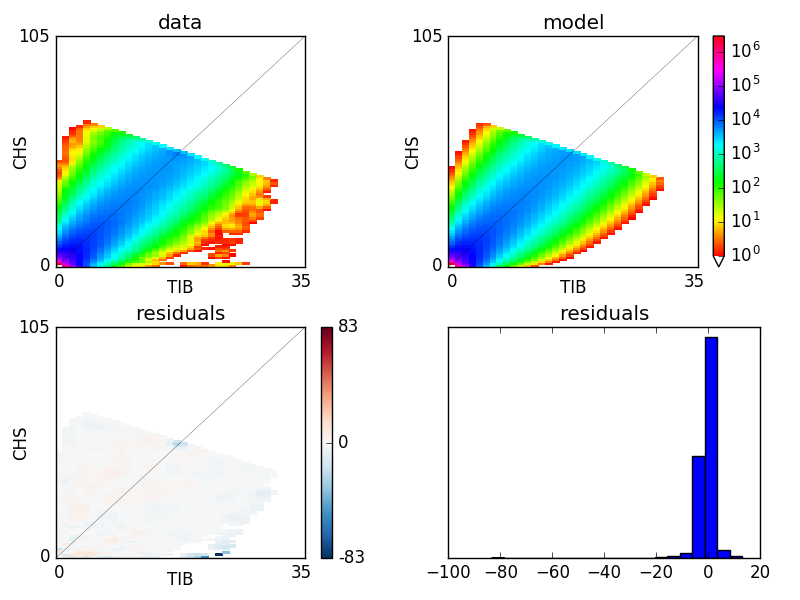


B.


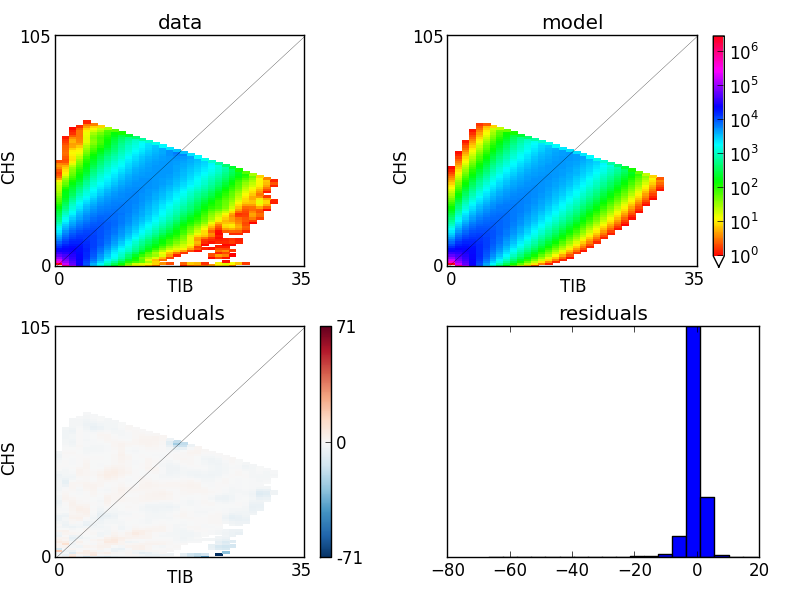


C.

D.

Supplement: S9 Fig — Panel A, B and C corresponds to ∂a∂i model A, B and C. In each panel, the first two plots show the observed and model predicted SFS heatmaps, respectively; the third plot shows the residual heatmap; the fourth plot shows a histogram of the residuals. Panel D shows the one-dimensional SFS for Han Chinese (left) and Tibetans (right) separately. Within each combination of population and model, the top plot shows the frequencies of variants for each minor allele count, with the red line showing the expected frequencies predicted by the model and blue line showing the observed frequencies; the bottom plot shows the standardized residuals of frequencies within each minor allele count category, assuming the frequencies are Poisson-distributed. (DOCX) [file pgen.1006675.s009.docx]
