# Supplementary material for: Evolutionary history of Tibetans inferred from whole-genome sequencing
Source: PLoS Genet. 2017 Apr 27;13(4):e1006675. doi: 10.1371/journal.pgen.1006675 (PMC5407610; doi:10.1371/journal.pgen.1006675)
Supplement: S1 Supplementary Methods — (DOCX) [file pgen.1006675.s021.docx]

**Supplementary Methods**

**Principal Component Analysis and recent admixture analysis**

To perform the Principal Component Analysis (PCA), we first combined Tibetan genomes with samples from 5 populations in the 1000 Genome Project: Yoruba, Han Chinese, Europeans, Punjabi and Peruvians. We included SNVs with minor allele frequencies greater than 5% and missing genotype calling rate less than 5% in the whole samples. After normalization of genotypes at SNV level, we used the prcomp() function in R to generate the principal components (PCs). Because PCA generates uncorrelated linear combinations of original variable, with earlier PCs explaining more variance in the data, the top 4 PCs were correlated with intercontinental separations of human populations rather than intracontinental separations. For example, PC1 separated the African population (Yoruba) from other populations; PC2 separated East Asian populations from Europeans; PC3 separated south American population (Peruvian) from Asians; PC4 separated Europeans from South Asians population (Punjabi). Due to their relative recent divergence time, Han and Tibetans were closely clustered in PC1 through PC4, but clearly separated on PC5.

We conducted the admixture analysis with the set of common variants used to conduct PCA. We conducted the analysis using the tool ADMIXTURE with a range of K values (from 2 to 8), which is the number of source populations. The cross-validation (CV) error rates were highest for K=2 and K=3 (0.560 and 0.536), but similar for K=4 (0.522), K=5 (0.522) and K=6 (0.524). For K=7 (0.527) and K=8 (0.531), the CV error rates were higher. From K=2 to K=5 we observed separations of major continental populations. At K=6, Tibetans and Han populations were distinct. We used the estimates from K=6 to identify Tibetan individuals with possible recent admixture from non-Tibetan populations; however, the admixture patterns within Tibetans were similar for higher K values.

**Estimating the demographic history of Tibetans with MSMC**

The multiple sequential Markovian coalescence (MSMC)[1] is an extension over the pairwise sequential Markovian coalescence (PSMC)[2] model. It allows for the estimation of effective population size in the population of interest, and the relative cross coalescence rates between two populations. The relative cross coalescence rates quantifies the genetic separation between populations, with a value close to 1 suggesting one single homogenous population and a value close to 0 suggesting complete separation. We used the MSMC master branch downloaded on May 1, 2015 for our study. Each individual genome was statistically phased with the shapeit2 package using 1000 Genome Project reference panel. For variants not present in the reference panel, we enumerated all possible phasing configurations in the MSMC input file. Because MSMC can analyze at most 4 diploid genomes at a time, we sampled 4 Han Chinese and 4 Tibetans with >99% corresponding genetic ancestry to estimate the history of effective population size. We used the --fixedRecombination parameter, as recommended by the online manual of MSMC. To estimate the relative cross coalescence rates (RCCR), we randomly selected 1 Han Chinese and 1 Tibetan genomes from the previous two sets. For comparison, we also computed the RCCR between the same Han genome and one European genome from 1000 Genomes Project (S8 Fig). We used the following parameters: “--fixedRecombination --skipAmbiguous -P 0,0,1,1”, as recommended. To generate bootstraps from the original data, we used the “multihetsep_bootstrap.py” script in the MSMC package.

**Estimating the demographic history of Tibetans with ∂a∂i**

We used the software package Diffusion Approximation for Demographic Inference, ∂a∂i[3] to construct the demographic history of Tibetans. Given a demographic history model with one or more free parameters, *∂a∂i* generates a maximum likelihood estimate for each parameter, including population sizes, event time, migration rates between populations, etc. Because we were mostly interested in the joint-demographic history of Han Chinese and Tibetans, we included in the analysis 56 Han Chinese and 19 Tibetan individuals who were predicted to have >99% corresponding genetic ancestries by ADMIXTURE. Complete Genomics Inc. (CGI) initially called the genotypes in-house and generated the VCF genotype files; we merged the two datasets using the vcf2cdr and cdr_manipulator tool from the VAAST software package, ensuring that missing genotype calls were retained in both datasets. We excluded SNVs that met any of the following criteria: 1) SNVs with >5% missing-genotype rate in our joint sample of CG 1000 Genomes data and Tibetans; 2) SNVs with >5% missing genotype rate in either 56 Han individuals or 19 Tibetan individuals; 3) SNVs with >5% discordant genotype calls between the CG public genome data and 1KG project Phase I data. We then used ∂a∂i to project the joint SFS to a sample size of 104 Han chromosomes and 34 Tibetan chromosomes, and to fold the SFS so that the inference is robust to mis-specified ancestral allele status. The final dataset contained 9,598,726 informative SNVs (i.e., SNVs not fixed in both samples with the same allele).

We first experimented with a relatively simple two-population separation model (model A). This model assumes an ancestral population with population size of nA1. After the out-of-African (OOA) event, the population size was reduced to nC1+nT1, which lasted for a time of T1. The two populations then separated at time T2 prior to present; since then, Han Chinese grew in effective population size from nC1 to nC2, and Tibetan grew in effective population size from nT1 to nT2. The migration rates between Han and Tibetan are symmetrically m1. We started 200 ∂a∂i runs with randomized initial parameters. The final converged model has a log-likelihood of -20373. This model predicted that Han and Tibetans split 39,000 years ago, with a migration rate of 3.7e-4 per generation per chromosome afterwards. (For all the calculations, we assumed an average generation time of 25 years, and a mutation rate of 1.2e-8 per nucleotide per generation. We listed the parameter boundaries for all three ∂a∂i models in S10 Table.)

Previously, Qi et al.[4] found evidence for both Upper Paleolithic and Neolithic migrations into the Tibetan Plateau. We wished to test whether our SFS data supports this finding. To do so, we revised the previous model A to construct a 2-stage separation-and-migration model (model B). This model was identical to model A except that Han and Tibetan first split at time T2+T3 before present. Since then, both populations exponentially grew for a time of T2 (period 1) with symmetric migration rates of m1, and then grew for a time of T3 (period 2) with symmetric migration rates of m2. During the two period the population growth rates were the same. The final converged model from 200 ∂a∂i runs has a log-likelihood of -11525; it predicts that the first period started 43,000 years ago and the second period started 12,000 years ago, with corresponding symmetric migration rates of 9.4e-4 and 1.79e-5 (per generation per chromosome).

Lastly, we relaxed two assumptions in the model above: (1) equal growth rates during the two periods and (2) symmetric migration rates between Han and Tibetan, by adding 4 additional parameters (2 parameters for different growth rates for the first and second periods, and 2 for the asymmetric migration rates between Han and Tibetans). This model (model C) is illustrated in details in Table 1 and Figure 2. The converged model from 200 ∂a∂i runs has a log-likelihood of -11174. We also presented the SFS comparisons between the observed data and model predictions in S9 Fig.

To calculate the confidence interval for the parameters in our best-fitting ∂a∂i model, we performed bootstrap sampling 100 times from all one-megabase genomic segments. For each bootstrap sample, we calculated SFS and ran ∂a∂i 50 times to estimate parameters of model C. The 2.5 to 97.5 percentile of parameter estimates from bootstraps was used as the corresponding 95% confidence interval.

Model C is a generalization of model A and B, and had substantially higher log-likelihood (-11174) compared to simpler models (-20373 and -11525). However, because the log-likelihoods calculated by ∂a∂i were composite log-likelihoods, standard likelihood ratio test and Akaiki Information Criterion for model selection were not applicable[3]. Instead, we examined the bootstrap confidence interval (CI) of key parameters in model C to determine if it was a substantially better fit than simpler models. Compared to model C, model B assumed that the population growth rate was the same between period 1 (from T2+T3 before present to T3 before present) and period 2 (from T3 before present to present). To test this assumption, we calculated the 95% CI of the ratio of population growth rate between period 2 and period 1; a ratio significantly different from 1 would provide evidence against the null assumption. For Han, the 95% CI was [1.67, 4.12]; for Tibetans, the 95% CI was [4.14, 14.46]. Therefore, in both populations, we were able to reject the equal population growth rate assumption. Model B also assumed symmetric migration rate from Han to Tibetan and from Tibetan to Han. We thus calculated the ratio of these two migration rates. In period 1, we found suggestive evidence against the symmetric migration rate assumption (the 95% CI of the ratio was [0.96, 1.70] and the 90% CI was [1.03,1.62]); in period 2, we cannot reject the null (95% CI: [1.56x10^-7^, 4.88x10^5^]) because of the large standard error of the Han to Tibetan migration rate estimate (Table 1). Lastly, we tested the following assumption of model A: the migration rates were the same between period 1 and period 2. The 95% CI for the ratio of period 1 versus period 2 migration rates from Tibetan to Han was [1.16x10^-8^, 2.83x10^-8^]; the same CI but from Han to Tibetan was [2.54x10^-15^, 6.87x10^-3^]. Therefore, in both cases we were able to reject the equal migration rates assumption. Considering all aforementioned evidences, we concluded that our data was most consistent with model C, which allowed for different and asymmetric migration rates between period 1 and 2, and different population growth rates between these two periods.

**Composite of Multiple Signals (CMS) test**

When we initiated the CMS test, two Han Chinese whole-genome sequencing datasets were publicly available: CG whole-genome sequencing data on the 1000 Genomes Project samples[5] and the 1000 Genomes Project Phase I genome data phased by Shapeit2[6]. The latter combined whole genome sequencing data with SNP microarray during phasing, and improved the genotype concordance rate as well as imputation performance[7]. Our haplotype-based selection tests (ΔiHH, XP-EHH and iHS) suggested that the Shapeit2 dataset tended to have longer extended haplotypes relative to the CG dataset and therefore led to fewer false-positive selective sweep signals. Yet, one limitation of the Shapeit2 dataset is that the genomic reads were a combination of high coverage exome sequencing and low coverage genomic sequencing data; therefore, some SNVs called in our Tibetan sampled were not present in the second control genome dataset. To remedy this, we performed CMS tests on both sets, and then combined the results with the following procedure. Whenever available, we used the CMS results using the Shapeit2 dataset as controls; if the focal SNV is absent, we used the CMS results on the CG set. This way, we maximized the quality of our CMS test while ensuring all SNVs that passed quality controls were scored.

One question in the implementation of CMS test was whether we should include the 8 Tibetans with moderate amount of recent admixture from other populations (>1%) in our test. Excluding these individuals may improve the signal-to-noise ratio for certain tests such as FST and ΔDAF, but at the cost of substantially reduce the overall sample size (from 27 to 19 genomes). To determine the optimal strategy, we performed coalescence simulations using the Tibetan demographic history predicted by ∂a∂i. We simulated 1000 1-megabase genomic regions containing a positively selected SNV (alternative model) and 1000 1-megabase genomic regions that do not contain a selected SNV (null model), and performed CMS tests in two different scenarios: 1) the cases were 38 Tibetans haplotypes with 100% Tibetan ancestry; 2) the cases were 54 Tibetan haplotypes, but each haplotype had 5.2% chance of being replaced by a Han haplotype (5.2% corresponded to the average proportion of foreign admixtures among Tibetans). In both scenarios, the controls were 100 Han Chinese haplotypes with no recent admixture. We plotted the distribution of CMS scores under null and alternative models in both scenarios in S10 Fig. Under the null model, the mean CMS score in scenario 1 (unadmixed) was slightly higher than scenario 2 (admixed) (-88.6 vs. -89.6), while under the alternative model, the mean CMS score in scenario 1 (unadmixed) was lower (-31.6 vs. -29.4). We note that a higher CMS score typically indicates a stronger signal of positive selection. This suggested that including the 8 Tibetans with recent admixture could actually improve the power of CMS test, likely due to the much higher sample size (a 42% increase). Therefore, we used all 27 Tibetans in our CMS tests.

**Genotyping the D4E (c.12C>G) and C127X (c.380G>C) variants in the *EGLN1* gene**

We previously identified two non-synonymous variants in the *EGLN1* gene (c.12C>G, p.D4E; c.380G>C, p.C127S) which together act as a co-adapted gene complex contributing to high-altitude adaptation in Tibetans. Both sites contained excessive missing genotype calls (>5%) and as a result failed our QC steps initially. Therefore, we performed additional Sanger sequencing on these two variants to generate their accurate genotypes. At c.12C>G, the number of Tibetans carrying the C/C, C/G and G/G genotypes were 3, 10 and 14, respectively. At c.380G>C, the number of Tibetans carrying the G/G, G/C and C/C genotypes were 3, 6 and 18, respectively.

**Identifying regions of Denisovan admixture using the S* statistic**

To fine-map Tibetan genomic regions with Denisovan admixture, we implemented the procedure developed by [8] utilizing the S* statistic. S* is a summary statistic designed to identify archaic haplotypes using linkage disequilibrium information[9]. In this study, we use 62 Han genomes from the 1000 Genome Project as the reference sequence, and one Denisovan genome[10] as the archaic sequence. We removed low-quality genotype calls and performed the haplotype phasing on the modern human genomes as described above.

We first calculated S* in all 50-kb genomic windows for each Tibetan genome. We removed variants present in Yoruba reference genomes and identified a subset of variants that maximized S* using the dynamic programming approach described in[8]. We repeated this process 5 times, each time randomly selecting 20 out of 27 Tibetan genomes to run S*, following the recommendations of [8]. The max S* score of each region from all replicates was recorded with the corresponding haplotype.

The distribution of S* values in our dataset was very similar to the published dataset, which is consistent with the previous report that the choices of S* cutoff were largely stable across different demographic models[8]. Therefore, we used an S* score cutoff of 10,000, which corresponded to an empirical p-value of approximately 0.1 in our study, to select the potential introgression regions. We further filtered the following regions as in [8]: (1) those overlapping known segmental duplications in the human genome[11]; (2) those with extremely high recombination rates; and (3) those where less than 80% of all non-reference SNVs in the target region were in the same chromosome. For every putative introgressed haplotype that passed these criteria, we evaluated their similarities to the Denisovan haplotypes, which were defined as the proportion of variants that were present in Denisovan, among all human and Denisovan variants in this region (M_n_). We calculated the empirical p-value of M_n_ by comparing it to the M_n_ distribution of similar haplotypes in non-introgressed portions of the genome. Here, “similar haplotypes” were defined as haplotypes within the similar number of variant sites (±5) and haplotype length (±1kb). We selected regions with empirical p-values less than 0.05 as our candidate introgressed regions.

**Estimating the adaptive *EPAS1* haplotype age and introgression date**

To account for potential biases in the estimated age of the selective sweep on the adaptive *EPAS1* haplotype, we employed a likelihood-based approach. Using the demographic model estimated by ∂a∂i, we performed msms coalescence simulations[12] on a grid of selection coefficients (from 0.005 to 0.045 with an interval of 0.001) and selective sweep start times (from 1 kya to 30 kya with an interval of 1000 years). We assumed an initial allele frequency of the selected allele of 0.004, matching the Denisovan introgression estimate. At each grid point, we generated 1 million simulations, and recorded the number of times where 1) the present-day allele frequency of the adaptive haplotype in the simulated samples exactly matched that of our Tibetan participants; and 2) the length of the adaptive haplotype needed to reduce homozygosity to 0.25 [13] was equal to 0.22 (+/- 0.01 cM), which was the length observed in our Tibetan participants. We estimated the likelihood for each grid point as the proportion of matches within 1 million simulations. We used the selective sweep start time that generated the maximum likelihood (L_max_) as our point estimate (). Furthermore, we searched in the neighborhood of for the lower and upper values where the likelihood dropped to, which formed the likelihood-ratio test confidence interval of selective sweep start time. This is because the true parameter *t* and the maximum likelihood estimate satisfy:

 (1)

where is a chi-square distribution with one degree of freedom[14].

To calculate an upper bound of the introgression date, we assumed that the amount of Denisovan haplotype introduced into the Tibetan population at the time of introgression is 0.4% on total Tibetan individuals, based on our point estimate of genome-wide Denisovan admixture proportion. We asked the question: in order to maintain a haplotype of length 0.249 cM (the distance between the first and last Denisovan-like variant on the *EPAS1* haplotype), what is the maximal amount of time that could have passed from introgression to the start of selection? To address this, we performed forward-time simulations with customized scripts, using the estimated Tibetan demographic history generated by ∂a∂i, and assuming exponential decay of haplotypes over time. When the time gap was 800 generations, the probability of having a ≥0.249 cM Denisovan haplotype surrounding the selective variant was 5%. Therefore, using the estimate that the adaptive selection starts 480 generations ago (calculated in the previous paragraph), the 95% upper bound of the estimated introgression date is 25*(480+800)=32,000 years ago. We also point out that this is a conservative estimate since the actual Denisovan haplotype length at the start of selection can be longer than 0.249cM.

We estimated the coalescence time of the *EPAS1* haplotype and the corresponding Denisovan genomic region by counting the number of divergent nucleotides. The most common Tibetan *EPAS1* haplotype was compared to homozygous sites in the same Denisovan region. The left and right boundaries of the *EPAS1* haplotype were set at Chr2:46552202 and Chr2:46791755 based on visual inspection of the Tibetan haplotype block (Figure 4b). We also experimented with smaller haplotype block sizes (150kb and 200kb), and found no significant difference in Tibetan-Denisovan nucleotide divergence rate (p=0.73 from χ^2^ test) within this region. Using the two smaller haplotype block sizes predicted divergence time of 989 kya and 1070 kya respectively, which were within the confidence interval given in the Result section. To eliminate the bias from natural selection and genotyping error, we removed exonic regions and sites where the genotype quality in the Denisovan sequence are below 30. We used a generation time of 25 years and a mutation rate of 1.2e-8 mutation per nucleotide per generation.

We calculated the Maximum Likelihood Estimate (MLE) and confidence interval of population divergence time *T_d_* (in generations) between Denisovan and the population contributing *EPAS1* haplotype to Tibetans (Denisovan-like).The likelihood of T_d_ is given by:

$\begin{matrix} L(T_{d}|n)=\int_{T_{d}}^{+\infty} \Pr(n,T_{g}|T_{d})dT_{g} \\ \text{ }=\int_{T_{d}}^{+\infty} \Pr(n|T_{g})\Pr(T_{g}|T_{d})dT_{g} \\ \text{ }=\int_{T_{d}}^{+\infty} \frac{\exp(-2\mu T_{g})(2\mu T_{g})^{n}}{n!}\Pr(T_{g}|T_{d})dT_{g} \end{matrix}$ (2)

where T_g_ is the *EPAS1* haplotype divergence time (in generations) between Tibetan and Denisovan; *n* is the number of divergent nucleotides at *EPAS1*; μ is the per generation mutation rate of the *EPAS1* locus. Pr(n|T_g_) is the probability of observing *n* mutations between Tibetan and Denisovan *EPAS1* haplotypes, which has a Poisson distribution. The value T_g_-T_d_ is the time to haplotype coalescence from the point of population divergence, which has an exponential distribution with the mean being 2N_e_, assuming that the ancestral population has a constant size of N_e_. However, if the ancestral population changed its size from N_1_ to N_2_ at time T_c_, then

$\Pr(T_{g}|T_{d})=\left\{\begin{matrix} 0\text{ if T}_{g}\text{<T}_{d} \\ \frac{1}{2N_{2}}\exp(-\frac{T_{g}-T_{d}}{2N_{2}})\text{ if }T_{d}\leq T_{g}\leq T_{c} \\ \exp(-\frac{T_{c}-T_{d}}{2N_{2}})\times\frac{1}{2N_{1}}\exp(-\frac{T_{g}-T_{c}}{2N_{1}})\text{ if }T_{g}>T_{c} \end{matrix}\right.$ (3)

In our calculation, we used the estimated population history of Denisovan from [10]. By (2) and (3), we can calculate the likelihood of any given T_d_ with numeric integrations. We used the T_d_ value that maximized L(T_d_|n) as our point estimate of T_d_. We used standard technique to calculate the 95% confidence interval of T_d_[14]. Namely, to calculate the lower bound, we found the T_d_ value such that:

$\sum_{k=1}^{n} L(T_{d}|n)=0.95$ (4)

To calculate the upper bound, we found the T_d_ value such that:

$\sum_{k=1}^{n-1} L(T_{d}|n)=0.05$ (5)

This gave us an MLE of 868 kya and 95% CI of 952 kya to 238 kya for the population divergence time between Denisovan and Denisovan-like populations.

1. Schiffels S, Durbin R (2014) Inferring human population size and separation history from multiple genome sequences. Nat Genet 46: 919-925.

2. Li H, Durbin R (2011) Inference of human population history from individual whole-genome sequences. Nature 475: 493-496.

3. Gutenkunst RN, Hernandez RD, Williamson SH, Bustamante CD (2009) Inferring the joint demographic history of multiple populations from multidimensional SNP frequency data. PLoS Genet 5: e1000695.

4. Qi X, Cui C, Peng Y, Zhang X, Yang Z, et al. (2013) Genetic evidence of paleolithic colonization and neolithic expansion of modern humans on the tibetan plateau. Mol Biol Evol 30: 1761-1778.

5. Genomes Project C, Abecasis GR, Auton A, Brooks LD, DePristo MA, et al. (2012) An integrated map of genetic variation from 1,092 human genomes. Nature 491: 56-65.

6. Delaneau O, Marchini J, Zagury JF (2012) A linear complexity phasing method for thousands of genomes. Nat Methods 9: 179-181.

7. Delaneau O, Marchini J, Genomes Project C, Genomes Project C (2014) Integrating sequence and array data to create an improved 1000 Genomes Project haplotype reference panel. Nat Commun 5: 3934.

8. Vernot B, Akey JM (2014) Resurrecting surviving Neandertal lineages from modern human genomes. Science 343: 1017-1021.

9. Plagnol V, Wall JD (2006) Possible ancestral structure in human populations. PLoS Genet 2: e105.

10. Meyer M, Kircher M, Gansauge MT, Li H, Racimo F, et al. (2012) A high-coverage genome sequence from an archaic Denisovan individual. Science 338: 222-226.

11. Bailey JA, Gu Z, Clark RA, Reinert K, Samonte RV, et al. (2002) Recent segmental duplications in the human genome. Science 297: 1003-1007.

12. Ewing G, Hermisson J (2010) MSMS: a coalescent simulation program including recombination, demographic structure and selection at a single locus. Bioinformatics 26: 2064-2065.

13. Voight BF, Kudaravalli S, Wen X, Pritchard JK (2006) A map of recent positive selection in the human genome. PLoS Biol 4: e72.

14. Casella G, Berger RL (2002) Statistical inference: Duxbury Pacific Grove, CA.
