# Supplementary material for: Evolutionary history of Tibetans inferred from whole-genome sequencing
Source: PLoS Genet. 2017 Apr 27;13(4):e1006675. doi: 10.1371/journal.pgen.1006675 (PMC5407610; doi:10.1371/journal.pgen.1006675)
Supplement: Supplementary file 15 [file pgen.1006675.s005.docx]

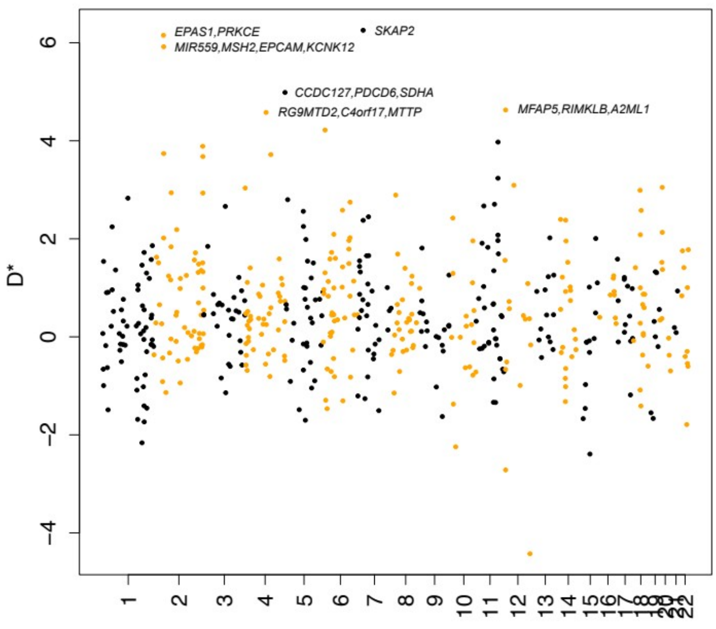

EPAS1,PRKCE
MIR559,MSH2,EPCAM,KCNK12
SKAP2
CCDC127,PDCD6,SDHA
RG9MTD2,C4orf17,MTTP
MFAP5,RIMKLB,A2ML1
D*
6
4
2
0
−2
−4
1
2
3
4
5
6
7
8
9
10
11
12
13
14
15
16
17
18
19
20
21
22
