# Supplementary material for: Evolutionary history of Tibetans inferred from whole-genome sequencing
Source: PLoS Genet. 2017 Apr 27;13(4):e1006675. doi: 10.1371/journal.pgen.1006675 (PMC5407610; doi:10.1371/journal.pgen.1006675)
Supplement: Supplementary file 16 [file pgen.1006675.s006.docx]

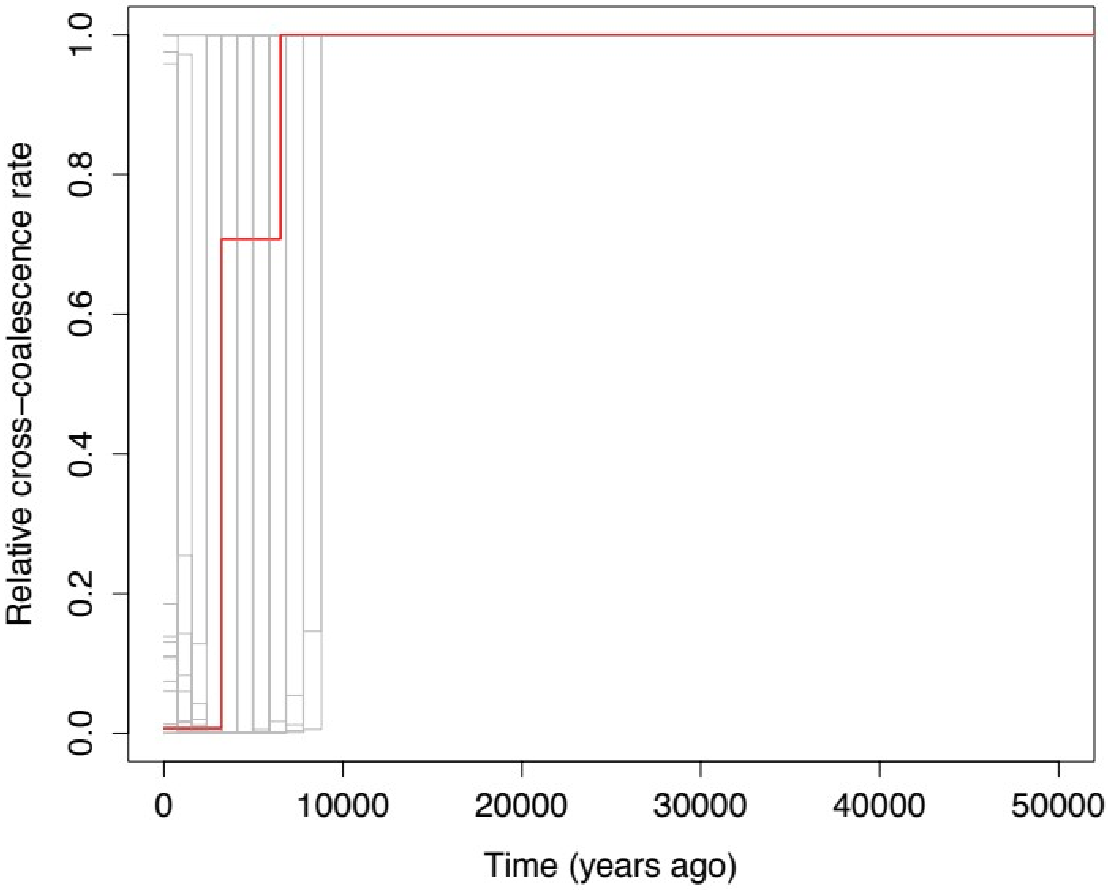

Relative cross−coalescence rate
0.0
0.2
0.4
0.6
0.8
1.0
0
10000
20000
30000
40000
50000
Time (years ago)
