# Supplementary material for: Evolutionary history of Tibetans inferred from whole-genome sequencing
Source: PLoS Genet. 2017 Apr 27;13(4):e1006675. doi: 10.1371/journal.pgen.1006675 (PMC5407610; doi:10.1371/journal.pgen.1006675)
Supplement: Supplementary file 17 [file pgen.1006675.s007.docx]

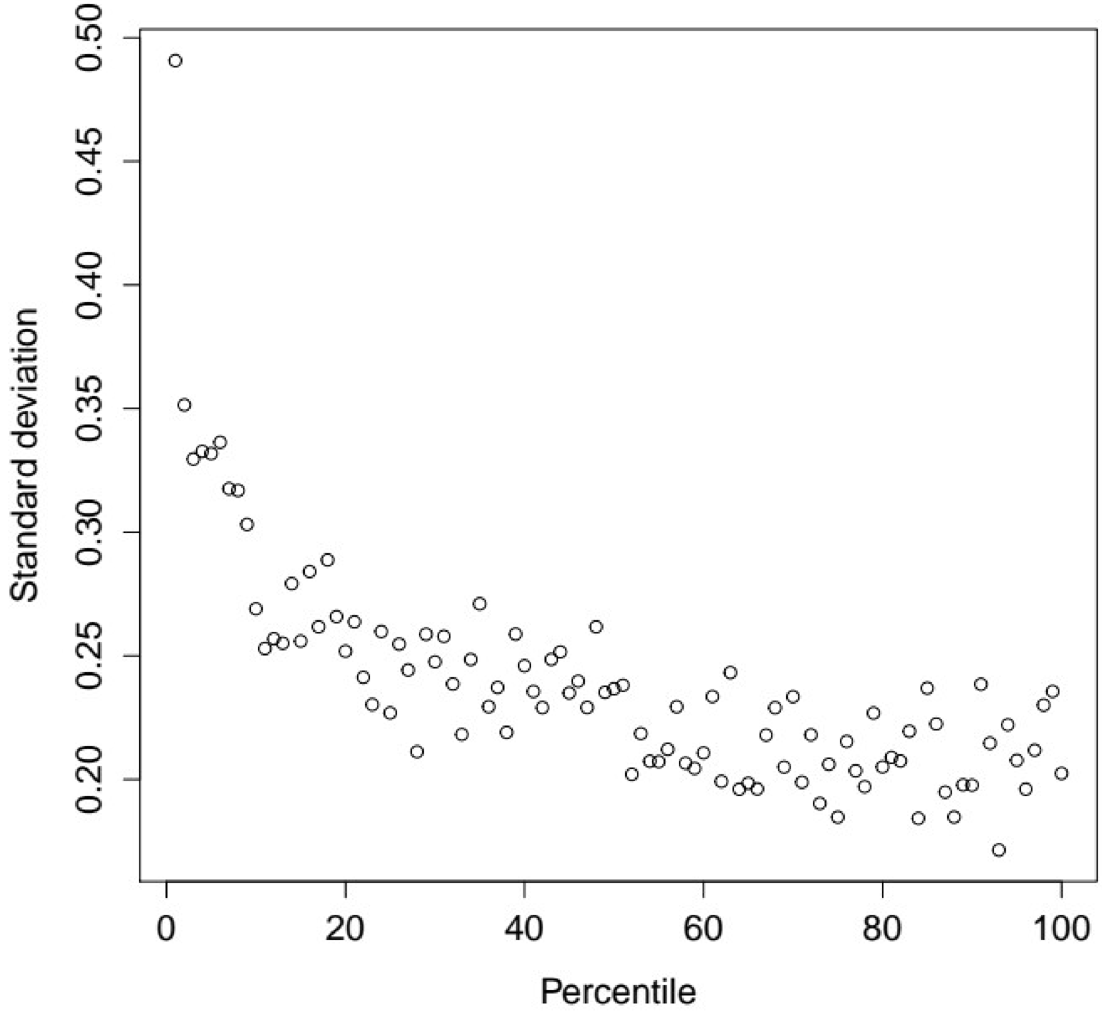

0.50
0.45
0.40
0.35
0.30
0.25
0.20
Standard deviation
0
20
40
60
80
100
Percentile
