# Supplementary material for: Evolutionary history of Tibetans inferred from whole-genome sequencing
Source: PLoS Genet. 2017 Apr 27;13(4):e1006675. doi: 10.1371/journal.pgen.1006675 (PMC5407610; doi:10.1371/journal.pgen.1006675)
Supplement: Supplementary file 18 [file pgen.1006675.s010.docx]

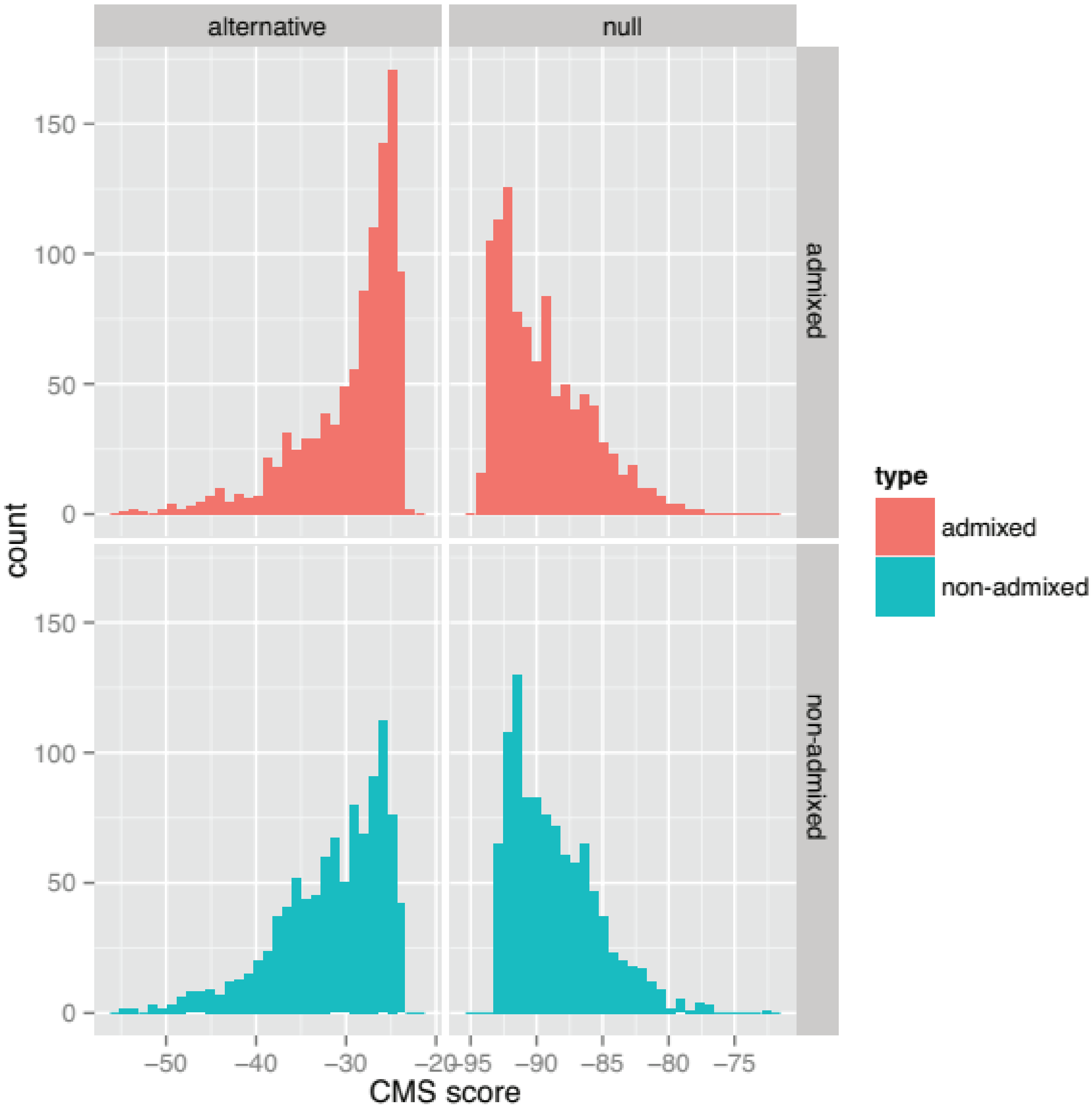

alternative
null
admixed
non-admixed
count
150
100
50
0
−50
−40
−30
−20
−95
−90
−85
−80
−75
CMS score
type
admixed
non-admixed
